# Development of a Robust *Saccharomyces cerevisiae* Strain for Efficient Co-Fermentation of Mixed Sugars and Enhanced Inhibitor Tolerance through Protoplast Fusion

**DOI:** 10.3390/microorganisms12081526

**Published:** 2024-07-25

**Authors:** Jianzhi Zhao, Yuping Zhao, Longhao Wu, Ning Yan, Shuo Yang, Lili Xu, Deyun He, Hongxing Li, Xiaoming Bao

**Affiliations:** Key Laboratory of Biobased Material and Green Papermaking, School of Bioengineering, Qilu University of Technology, Shandong Academy of Sciences, 3501 Daxue Road, Jinan 250353, China; zhaojianzhi@qlu.edu.cn (J.Z.); zhaoyuping0718@126.com (Y.Z.); wuloonghao@outlook.com (L.W.); cathyyann@163.com (N.Y.); yangshuo974@163.com (S.Y.); xulili@qlu.edu.cn (L.X.); hedeyun@qlu.edu.cn (D.H.); baoxm@qlu.edu.cn (X.B.)

**Keywords:** protoplast fusion, strain screening, inhibitor tolerance, cellulosic ethanol, C5/C6 co-fermentation, *Saccharomyces cerevisiae*

## Abstract

The economical and efficient commercial production of second-generation bioethanol requires fermentation microorganisms capable of entirely and rapidly utilizing all sugars in lignocellulosic hydrolysates. In this study, we developed a recombinant *Saccharomyces cerevisiae* strain, BLH510, through protoplast fusion and metabolic engineering to enhance its ability to co-ferment glucose, xylose, cellobiose, and xylooligosaccharides while tolerating various inhibitors commonly found in lignocellulosic hydrolysates. The parental strains, LF1 and BLN26, were selected for their superior glucose/xylose co-fermentation capabilities and inhibitor tolerance, respectively. The fusion strain BLH510 demonstrated efficient utilization of mixed sugars and high ethanol yield under oxygen-limited conditions. Under low inoculum conditions, strain BLH510 could completely consume all four kinds of sugars in the medium within 84 h. The fermentation produced 33.96 g/L ethanol, achieving 84.3% of the theoretical ethanol yield. Despite the challenging presence of mixed inhibitors, BLH510 successfully metabolized all four sugars above after 120 h of fermentation, producing approximately 30 g/L ethanol and reaching 83% of the theoretical yield. Also, strain BLH510 exhibited increased intracellular trehalose content, particularly under conditions with mixed inhibitors, where the intracellular trehalose reached 239.3 mg/g yeast biomass. This elevated trehalose content contributes to the enhanced stress tolerance of BLH510. The study also optimized conditions for protoplast preparation and fusion, balancing high preparation efficiency and satisfactory regeneration efficiency. The results indicate that BLH510 is a promising candidate for industrial second-generation bioethanol production from lignocellulosic biomass, offering improved performance under challenging fermentation conditions. Our work demonstrates the potential of combining protoplast fusion and metabolic engineering to develop superior *S. cerevisiae* strains for lignocellulosic bioethanol production. This approach can also be extended to develop robust microbial platforms for producing a wide array of lignocellulosic biomass-based biochemicals.

## 1. Introduction

Fuel ethanol production offers significant environmental, economic, and energy security benefits. It reduces greenhouse gas (GHG) emissions, supports rural economies, and enhances energy security by diversifying fuel sources [1].

Lignocellulosic ethanol, also known as cellulosic ethanol or second-generation (2G) ethanol, represents a promising and sustainable alternative to traditional fossil fuels and first-generation (1G) biofuels. This 2G biofuel is produced from lignocellulosic biomass (LCB), which includes agricultural residues (e.g., corn stover, wheat or rice straw, sugar cane bagasse), forestry waste (e.g., wood chips, sawdust), and dedicated energy crops (e.g., switchgrass, miscanthus) [2]. This diversity and abundance of feedstock ensures a stable and sustainable supply for ethanol production without competing with food crops, thus avoiding the “food vs. fuel” dilemma associated with 1G biofuels like corn ethanol [3].

Despite its potential, the commercialization of 2G ethanol faces several technical challenges associated with efficient and economical conversion [2,4]. LCB primarily consists of cellulose, hemicellulose, and lignin, with the specific proportions of these components varying based on the plant biomass source [5,6,7]. This complex polymeric structure is inherently resistant to enzymatic degradation, necessitating pretreatment processes to enhance the efficiency of subsequent enzymatic hydrolysis [8]. The complex pretreatment and hydrolysis process leads to high production costs, a significant barrier to the economic feasibility of 2G ethanol.

The hexose (C6) and pentose (C5) sugars produced from the enzymatic hydrolysis of polymeric carbohydrates are commercially fermented into ethanol using engineered yeast, bacterial, and fungal communities [2,9]. Among these, the budding yeast *Saccharomyces cerevisiae* stands out as a critical microorganism in industrial biotechnology, and its inherent advantages have solidified its position as the preferred choice for traditional bioethanol production [10,11]. Moreover, the versatility of *S. cerevisiae* extends beyond conventional applications, as it has become a cornerstone in producing advanced fuels and chemicals derived from LCB feedstocks [11,12]. For the economical and efficient production of 2G ethanol, it is crucial to achieve complete and rapid utilization of all sugars present in the hydrolysates derived from LCB [11,13]. This necessitates several specific requirements for fermentation microorganisms such as *S. cerevisiae*. First, the microorganism must be able to co-ferment both C6 and C5 sugars (particularly D-xylose, the second most abundant sugar in LCB hydrolysate), while natural *S. cerevisiae* typically lacks the ability to metabolize C5 sugars [14]. Second, given the individual and synergistic negative interactions derived from the various inhibitory compounds that are formed during the pretreatment and hydrolysis stages of the LCB hydrolysate production [15], *S. cerevisiae* must exhibit robust tolerance to mitigate the adverse effects of these inhibitors on 2G ethanol fermentation [11]. Additionally, LCB hydrolysate often contains a certain amount of oligosaccharides, including cellobiose and xylooligosaccharides (XOS). Cellobiose is a disaccharide of two glucose molecules linked by a β-1,4-glycosidic bond. It is a significant intermediate in the enzymatic hydrolysis of cellulose and is commonly found in LCB hydrolysate, particularly when the β-glucosidase (hydrolyzing cellobiose to glucose, BGL) activity is insufficient to hydrolyze cellobiose into glucose fully. Notably, despite its widespread industrial applications, the cellulase system of filamentous fungi such as *Trichoderma reesei* typically exhibits limited β-glucosidase activity [16]. Furthermore, cellobiose acts as a potent inhibitor of cellobiohydrolase (cleaving cellulose from reducing or nonreducing ends to release cellobiose, CBH), which are crucial for cellulase activity [17]. Xylooligosaccharides (XOS), consisting of 2–7 xylose units linked by β-1,4-glycosidic bonds, are produced from the hydrolysis of xylan, a major hemicellulose component. To maximize xylose yield and minimize inhibitor production, milder pretreatment methods are often adopted, resulting in residual xylan and XOS in the hydrolysate [18]. Cellobiose and XOS are known to impede cellulose hydrolysis efficiency by cellulases significantly. Recent studies have revealed that this inhibition persists even with increased cellulose substrate or cellulase loading [19]. However, most commercially available cellulases are deficient in β-xylosidase activity, which is crucial for the complete hydrolysis of XOS. To circumvent the costly supplementation of exogenous β-glucosidase and β-xylosidase [17], a promising strategy involves the heterologous expression of these enzymes in fermentation microbes such as *S. cerevisiae* [19]. This approach not only alleviates the inhibitory effects of cellobiose and XOS but also enables the direct fermentation of the released sugars, streamlining the overall process and potentially reducing production costs.

Extensive efforts have been devoted to enhancing the xylose metabolism capacity of engineered *S. cerevisiae*. These efforts include engineering the XR/XDH (xylose reductase and xylitol dehydrogenase) or XI (xylose isomerase) pathway, optimizing cofactors, modifying transporters, and employing evolutionary engineering strategies [20,21,22,23,24]. In our previous study, we developed the engineered strain *S. cerevisiae* LF1, recognized as an outstanding xylose-utilizing strain, through rational metabolic engineering and adaptive evolution techniques. This strain exhibited a remarkable capacity to co-ferment a mixture of 80 g/L glucose and 40 g/L xylose in a synthetic medium. Within a mere 16 h, the strain completely consumed both sugars, achieving an impressive ethanol yield of 0.48 g/g total sugars [11,25]. This yield approaches the theoretical maximum, highlighting the strain’s efficient conversion of the sugar mixture to ethanol.

Despite the remarkable xylose fermentation capacities demonstrated by the current recombinant *S. cerevisiae* strains in synthetic media, their conversion efficiency toward LCB hydrolysates remains insufficient due to the synergistic adverse effects of multiple inhibitors generated during LCB pretreatment, even though many rational strategies, including genetic manipulation of relevant oxidoreductase genes, transcription factors, and regulation of purine biosynthetic pathway expression, have been effectively applied to improve individual inhibitor tolerance over the past decade [25,26,27,28]. Additionally, irrational strategies, including genome shuffling, random mutagenesis, and adaptive evolutionary engineering, have been employed to improve tolerance to multiple inhibitors [29,30]. However, most research has focused on yeast tolerance to individual inhibitors [25], and antagonism between high xylose utilization and robustness has been observed in reported xylose-utilizing strains [11,31,32]. In summary, developing *S. cerevisiae* strains with the combined capabilities of glucose/xylose co-metabolism, inhibitor tolerance, and oligosaccharide hydrolysis is critical for the economical and efficient production of 2G ethanol. 

In our previous study, strain 6M-15 with improved xylose fermentation performance and significantly enhanced tolerance to multiple inhibitors was obtained by combining iterative ARTP mutagenesis and anaerobic screening [25]. This demonstrates the feasibility of engineering yeast strains to alleviate the antagonism between high xylose utilization and robustness. A recombinant haploid *S. cerevisiae* strain BSGIBX was constructed in which the xylose isomerase gene Ru-*xylA* (isolated from a bovine rumen metagenomics library and denoted as “Ru” to indicate its rumen origin) and the β-xylosidase cassette IBX (β-xylosidase gene *xyl3A* from *Penicillium oxalicum* fused with the signal peptide of *Kluyveromyces INU*) were successfully co-expressed. BSGIBX can grow and produce ethanol using XOS as the sole carbon source, providing theoretical support for our work on robust industrial yeast strains [19]. Then, after a comprehensive screening process, *S. cerevisiae* RC212 was selected as the chassis strain for multi-copy integration of heterologous β-glucosidase and β-xylosidase genes [12]. The recombinant strain, BLN26, was paired with the previously engineered strain LF1 to form a binary system. This innovative dual-strain approach enabled the partial fermentation of four distinct sugars: glucose, xylose, cellobiose, and XOS. 

Protoplast fusion technology is a powerful tool in biotechnology; it offers numerous advantages and has a wide range of applications in improving strain performance across various organisms. Its ability to create novel genetic combinations and overcome traditional breeding limitations makes it a valuable tool in modern biotechnology. Many research works have achieved satisfactory results using protoplast fusion technology, such as increasing the production of various metabolites such as ethanol [33,34], organic acid [35], and antibiotics [36] and improving biodegradation [37] or tolerance to environmental stresses [38,39]. Building on the explanation mentioned above and our previous work, we selected LF1, an engineered strain with excellent glucose/xylose co-fermentation capabilities [11], and BLN26, an engineered *S. cerevisiae* strain with superior inhibitor tolerance and oligosaccharide hydrolysis abilities [12], as parental strains for protoplast fusion. We conducted rigorous screening and verification of the resulting fusion products after the fusion. We aim to develop a fusion strain that excels in glucose/xylose co-fermentation, exhibits high inhibitor tolerance, and possesses robust oligosaccharide hydrolysis and fermentation capabilities. Such a strain would significantly mitigate the antagonistic effects between high xylose utilization and robustness, making it a promising candidate for large-scale production of second-generation (2G) ethanol. This study utilized bioethanol as a reporter molecule to evaluate mixed-sugar assimilation competence. We anticipate that this *S. cerevisiae* fusant can serve as a versatile microbial platform to produce a wide array of lignocellulosic biomass (LCB)-based biochemicals.

## 2. Materials and Methods

### 2.1. Microbial Strains and Media

The genetic characteristics of the microbial strains employed and engineered in this study are summarized in Table 1. *Escherichia coli* Trans5α (TransGen, Beijing, China) served as the host strain for the amplification of recombinant plasmid in Luria–Bertani (LB) medium, which consisted of 5 g/L yeast extract (Oxoid, Basingstoke, UK), 10 g/L tryptone (Oxoid, Basingstoke, UK), and 10 g/L NaCl, with the pH adjusted to 7.0. When required, ampicillin (Sigma-Aldrich, St. Louis, MO, USA) was supplemented at a concentration of 100 mg/L.

All yeast strains were precultured at 30 °C in either YEPD medium, containing 10 g/L yeast extract, 20 g/L tryptone, and 20 g/L glucose, or YEPX medium, composed of 10 g/L yeast extract, 20 g/L tryptone, and 20 g/L xylose. G418 (Genview, Houston, TX, USA) was added at an appropriate concentration when necessary to maintain the selection pressure for recombinant strains. The corresponding solid medium was prepared by adding 20 g/L agar (Solarbio Science & Technology Co., Ltd., Beijing, China). Glucose (analytical reagent, AR) and xylose (AR) used in the culture medium were purchased from Sinopharm Chemical Reagent Co., Ltd. (Shanghai, China). Unless otherwise stated, all other chemicals used in this study were of analytical grade and commercially available.

The protoplast regeneration medium (RM) was prepared by adding 0.8 M sorbitol (MP Biomedicals Co., Ltd., Shanghai, China) to YEPD solid medium or YEPX solid medium to maintain a high osmotic pressure environment, reaching YPDS or YPXS medium, respectively. The YPXB solid medium was prepared by supplementing the YEPX solid medium with 20 ng/μL benomyl (Sigma-Aldrich, St. Louis, MO, USA) as one of the stress conditions to screen for fusant cells with stable genetic traits. The medium was sterilized by autoclaving at 115 °C for 30 min. 

The selection medium (SM) was based on YEPX and supplemented with various inhibitory compounds, including 10 mM acetic acid, 5 mM formic acid, 5 mM levulinic acid, 5 mM furfural, 5 mM HMF, and 5 mM vanillin, to facilitate the selection of fusants with improved tolerance to these stressors [40]. The above inhibitory compounds were purchased from Shanghai Aladdin Biochemical Technology Co., Ltd. (Shanghai, China). The medium was sterilized by autoclaving at 121 °C for 15 min, and the inhibitory compounds were added aseptically after cooling. The corresponding solid medium was prepared by adding 20 g/L agar. 

YP medium (10 g/L yeast extract, 20 g/L tryptone) was supplemented with various sugars as carbon sources for oxygen-limited growth and fermentation experiments. The medium was designated according to the specific sugar combinations employed: YPC for 20 g/L cellobiose (AR, Sinopharm Chemical Reagent Co., Ltd., Shanghai, China), YPGC for a mixture of 20 g/L glucose and 20 g/L cellobiose, YPXC for a mixture of 20 g/L xylose and 20 g/L cellobiose, and YPGXC for a mixture of 20 g/L glucose, 20 g/L xylose and 20 g/L cellobiose. When the carbon source consisted of a mixture of glucose–xylose–cellobiose and 20 g/L XOS (Shanghai Yuanye Biotechnology Co., Ltd., Shanghai, China) pretreated with xylanase, the medium was denoted as YPGXCO_pre_.

**Table 1:** Microbial strains and plasmids employed in the current study.

Strains and Plasmids	Description	Source/Reference
Strains		
CEN.PK102-5B	Haploid *S. cerevisiae* strain MATa; *URA*3-52, *HIS3*Δ1, *LEU2*-3, 112	Laboratory reserved [41]
LF1	Recombinant glucose/xylose co-fermenting strain derived from BSIF (*pho13::*XI, *3δ::*XI, *gre3::*PPP, XK, AE-PCS, *N360F*, AE)	Laboratory preserved [11]
BLN26	RC212 derivative; (*δ::*BGL& KanMX, *ADH2::*IBX, *δ::*IBX)	Laboratory preserved [12]
BLH01	Protoplast fusion strain from LF1 and BLN26	This work
BLH02	Protoplast fusion strain from LF1 and BLN26	This work
BLH172	Protoplast fusion strain with stable inheritance of traits	This work
BLH508	BLH172 derivative; *adh2*Δ*::*BGL	This work
BLH510	BLH508 derivative; *adh2*Δ*::*BGL, *δ::*IBX	This work
Plasmids		
YEp-CH	YEp24 derivative; *GAL1p*-*Cre*-*CYC1t*, *TEF1p*-*hygB*-*TEF1t*	Laboratory preserved [11]
pUG6	*E. coli* plasmid with segment loxP-KanMX4-loxP	Laboratory preserved [42]
pJXIHIBX	pJXIH-PC with β-xylosidase gene *xyl3A* from *P. oxalicum*; signal peptide of *Kluyveromyces INU*	Laboratory preserved [19]
pUCδBK	pUC19-based yeast integration plasmid containing the δ-sequence-targeting recombinant arms, an expression cassette of BGL, and the selectable marker loxP-KanMX4-loxP	Laboratory preserved [12]

### 2.2. Construction of Plasmids and Integrating Fragments

The plasmids used and constructed in this study are summarized in Table 1, along with their characteristics and applications. Table 2 provides a comprehensive list of the PCR primers employed in amplifying and cloning the target genes and genetic elements. Using plasmids pUCδBK and pUG6 as templates, we amplified the BGL fragment and the loxP-KanMX-loxP fragment via PCR with primers BGL-F/BGL-R and loxP-F/loxP-R, respectively. Additionally, using the BLH172 genome as template, we amplified the *ADH2* (alcohol dehydrogenase 2 gene) homologous fragments UP1adh2, UP2adh2, and DOWNadh2 with primers U1-ADH2-F/U1-ADH2-R, U2-ADH2-F/U2-ADH2-R, and D-ADH2-F/D-ADH2-R, respectively. The amplified fragments by overlapping PCR were purified, resulting in UP1adh2-BGL-loxP-KanMX-loxP-DOWNadh2 and UP2adh2-BGL-loxP-KanMX-loxP-DOWNadh2. The construction process of the integration fragments and the relative positions of the homology arms at the *ADH2* site are shown in Figure S1.

Using plasmids pJXIHIBX and pUG6 as templates, we amplified the IBX fragment and the loxP-KanMX-loxP fragment via PCR with primers XYL-F/XYL-R and loxP-F/loxP-R, respectively. Additionally, using the BLH508 genome as a template, we amplified δ1, δ2, and δ3 with primers UP1-δ-F/UP1-δ-R, UP2-δ-F/UP2-δ-R, and D-δ-F/D-δ-R, respectively.

### 2.3. The Preparation, Inactivation, and Fusion of Yeast Protoplasts

#### 2.3.1. Protoplasts Preparation

The parental strains, LF1 and BLN26, were activated separately in 5 mL of YEPD liquid medium for 12 h. They were then transferred to 40 mL of fresh YEPD liquid medium with an initial OD_600_ of 0.2 and cultured until the early logarithmic growth phase was reached, at which point the OD_600_ value was approximately 0.6~1.0. The cultures were centrifuged at 4500 rpm for 15 min to collect the cells, which were then washed twice with 20 mL of sterile water.

Subsequently, the cells were resuspended in 1 mL ddH_2_O (double-distilled water), the OD_600_ value was measured, and the final concentration of the cell suspension was adjusted to approximately 10^7^ cells/mL. To the cell suspension, 3 mL of an aliquoted sample containing 0.2% (*v*/*v*) β-mercaptoethanol and Tris-EDTA (TE) buffer was added, mixed well, and incubated at 30 °C for 20 min. The TE buffer was made up of 10 mM Tris-HCl (tris(hydroxymethyl)aminomethane hydrochloride, pH 8.0) and 1 mM EDTA (ethylenediaminetetraacetic acid). The cell suspension was centrifuged and resuspended in 4 mL of protoplast preparation reagent and 2 mL of S buffer containing different concentrations of yeast lytic enzyme Zymolyase 20T (the concentrations of which were 0.5, 1, 2, 3, 5 U/g (wet cell weight), respectively) for enzymatic digestion of the cell wall and incubated at 30 °C for 15 min. The S buffer comprised 1.0 M sorbitol, 10 mM PIPES (piperazine-N,N′-bis(2-ethanesulfonic acid)), pH 6.5. Then, the protoplasts were obtained by centrifugation at 5000 rpm for 5 min at 4 °C, followed by washing twice with S buffer. The chemical reagents β-mercaptoethanol, Tris-HCl, EDTA, sorbitol, PIPES, and Zymolyase 20T were purchased from MP Biomedicals Co., Ltd. (Shanghai, China).

A 1 μL sample was mixed with 20 μL of S buffer or 20 μL of ddH_2_O and observed under a microscope. If the protoplasts burst in water and remained intact in the S buffer, the preparation of protoplasts was considered successful.

#### 2.3.2. Protoplasts Inactivation

Heat inactivation of LF1 protoplasts: The protoplast suspension of the parental strain LF1 was transferred to sterile test tubes and incubated in a 60 °C water bath for 2, 5, 10, 15, and 20 min to determine the optimal inactivation conditions. The inactivation effect was confirmed by the inability of the treated protoplasts to grow on the regeneration medium (agar concentration of 0.8%).

Ultraviolet (UV) radiation inactivation of BLN26 protoplasts: The protoplast suspension of the parental strain BLN26 was transferred to sterile culture dishes with a diameter of 3 cm and placed on a preheated magnetic stirrer. The dishes were then exposed to a 30-watt UV lamp (with no other light sources) at a vertical distance of 20 cm for 1, 2, 5, 7, and 10 min to determine the optimal inactivation conditions. The treated protoplasts were kept in the dark for 2 h to prevent photoreactivation repair. The inactivation effect was confirmed by the inability of the treated protoplasts to grow on the regeneration medium (agar concentration of 0.8%).

#### 2.3.3. Protoplasts Fusion

The suspensions of inactivated parental protoplasts were adjusted to a concentration of approximately 1 × 10^7^ per mL. One mL of each suspension was centrifuged at 3000 rpm for 10 min at 4 °C. Two mL of polyethylene glycol 6000 (PEG 6000, BBI Life Sciences Co., Ltd., Shanghai, China) solution (preheated to 30 °C) was used to collect the parental protoplasts, which were then mixed thoroughly.

The mixed protoplasts were incubated in a 30 °C water bath for 20 min, followed by centrifugation at 3000 rpm for 10 min. The supernatant was discarded, and the pellet was resuspended in 2 mL of S buffer. The fused protoplasts were then regenerated on RM (protoplast regeneration medium). 

The protoplast fusion efficiency was calculated using the following formula:

Protoplast fusion efficiency (%) = [(A − B)/C] × 100%,

where A corresponds to the number of colonies observed on the RM (protoplast regeneration) medium YPXS, B corresponds to the number of colonies of the inactivated parents observed on RM medium YPDS, and C represents the number of parental colonies observed on YPDS.

### 2.4. The Screening of Yeast Fusants Derived from Protoplasts Fusion

Primary screening of fusants: Single colonies from the regeneration plates were transferred to 96-well plates containing 200 μL of YEPD liquid medium and incubated at 30 °C for 12 h in a microplate shaker incubator. The cultures were then spotted onto SM agar plates using a multi-channel pipette and incubated at 30 °C for 2–3 days. Thicker colonies were selected for secondary screening.

Secondary screening of fusants: The selected fusants and parental strains LF1 and BLN26 were activated as described above. They were then inoculated at an initial OD_600_ of 3.5 into 40 mL of YP medium containing either 40 g/L xylose (YPX40), 40 g/L glucose and 2× inhibitor mixture (YPG40I_2×_, 20 mM acetic acid, 10 mM formic acid, 10 mM levulinic acid, 10 mM HMF, 10 mM furfural, and 10 mM vanillin) or 40 g/L xylose and 1.5× inhibitor mixture (YPX40I_1.5×_, 15 mM acetic acid, 7.5 mM formic acid, 7.5 mM levulinic acid, 7.5 mM HMF, 7.5 mM furfural, and 7.5 mM vanillin). The inhibitory compounds were purchased from Shanghai Aladdin Biochemical Technology Co., Ltd. (Shanghai, China). Oxygen-limited fermentation was performed at 30 °C and 200 rpm. Samples were taken regularly to measure OD_600_, and the supernatants were analyzed by HPLC to determine the concentrations of metabolic products.

To avoid the influence of heterokaryosis after protoplast fusion, the fusants were continuously subcultured in 40 mL of liquid SM medium for multiple generations to ensure the stable inheritance of the desired traits. The fusants were then transferred to 40 mL of YEPD liquid medium and subcultured without selection pressure to confirm the stable transmission of the fusant characteristics.

### 2.5. Ploidy Determination of S. cerevisiae

The ploidy of the *S. cerevisiae* strains was determined by flow cytometry, using fluorescently labeled cell nuclei to analyze the DNA content of individual cells within the population. The haploid strain CEN.PK 102-5B served as a reference for comparison. *S. cerevisiae* cells were fixed in 70% (*v*/*v*) ethanol at a concentration of 2 × 10^6^ cells/mL and stored at –20 °C for 2 h. Following fixation, the cells were washed twice with phosphate-buffered saline (PBS; 137 mM NaCl, 2.7 mM KCl, 10 mM phosphate buffer, pH 7.4) and resuspended in 1 mL PBS containing 1 mg/mL RNAase A (Sigma-Aldrich, St. Louis, MO, USA). The cell suspension was incubated at 37 °C for 2 h to digest cellular RNA. After incubation with RNAase A, the cells were washed with PBS and resuspended in 200 μL of PBS containing 50 μg/mL propidium iodide (PI, Sigma-Aldrich, St. Louis, MO, USA), a fluorescent dye that intercalates into double-stranded DNA. The fluorescently stained cells were analyzed using a flow cytometer, with data collected on a linear scale. Under these conditions, the fluorescence intensity is directly proportional to the DNA content of each cell [43,44]. By comparing the fluorescence profiles of the test strains to that of the haploid reference strain CEN.PK 102-5B, the ploidy of the *S. cerevisiae* strains can be determined.

### 2.6. Oxygen-Limited Growth and Fermentation

Preculture and inoculum preparation: *S. cerevisiae* strains were initially grown in an appropriate volume of YEPD medium under aerobic conditions at 30 °C overnight. The overnight cultures were then transferred into fresh YEPD medium at an initial OD_600_ of 0.2 and cultured overnight to obtain the inoculum for fermentation experiments.

Fermentation setup: The prepared inoculum was withdrawn and inoculated into the fermentation medium, which was contained in 120 mL serum bottles. To create an oxygen-limited environment, the serum bottles were sealed with rubber stoppers, and a syringe needle was inserted through the stopper to allow for limited gas exchange. The initial cell density was adjusted to either OD_600_ = 0.2 or OD_600_ = 3.5, depending on the specific experimental requirements. All shake flask experiments were conducted on an orbital shaker set at 200 rpm. This agitation rate ensures proper mixing of the fermentation medium and facilitates the limited oxygen transfer necessary for oxygen-limited growth and fermentation.

Sampling and monitoring: Throughout the fermentation process, samples were collected regularly to monitor cell growth, substrate consumption, and product formation. The sampling frequency was determined based on the expected fermentation kinetics and the desired resolution of the data.

### 2.7. Determination of Intracellular Trehalose Content

The intracellular trehalose content of *S. cerevisiae* cells was determined following a specific cultivation protocol. Initially, the yeast cells were precultured and then inoculated into 50 mL of YEPD medium at an initial OD_600_ of 0.5. The cultures were incubated at 30 °C with shaking at 200 rpm for 12 h. Subsequently, the culture temperature was gradually increased by 1 °C every 21 min until it reached 37 °C. At this point, samples were collected to determine intracellular trehalose content. The quantification of trehalose was performed using the anthrone–sulfuric acid method, a colorimetric assay that relies on the reaction between trehalose and anthrone reagent in the presence of sulfuric acid [12,45]. A standard curve was prepared using known concentrations of trehalose to enable accurate quantification of the samples. To ensure the reliability and reproducibility of the results, each experimental group was performed in triplicate.

### 2.8. Determination of the Ratio of Reduced Glutathione (GSH) to Oxidized Glutathione (GSSG)

The ratio of reduced glutathione (GSH) to oxidized glutathione (GSSG) was determined using the GSH and GSSG assay kit (S0053, Beyotime Biotechnology, Shanghai, China). The specific experimental procedures and precautions were carried out in strict accordance with the manufacturer’s instructions provided with the kit. *S. cerevisiae* cells were cultured in YEPD medium with an initial OD_600_ of 0.2. The cells were harvested when the OD_600_ reached 4.0. After collection, the cells were washed with PBS buffer to remove residual medium components. To extract the glutathione, the cell mixture was combined with three times the volume of deproteinization buffer M, which was supplied as part of the S0053 assay kit. The samples were then subjected to two cycles of rapid freezing in liquid nitrogen followed by thawing in a 37 °C water bath. This process, known as multi-gelation, facilitates the efficient lysis of the cells and the release of intracellular glutathione. After the multi-gelation step, the samples were centrifuged at 13,000 rpm for 15 min at 4 °C. The resulting supernatant, containing the extracted glutathione, was carefully collected for subsequent GSH and GSSG determination. To ensure the reliability and reproducibility of the results, each experimental group was performed in triplicate.

### 2.9. Assays of β-Glucosidase, β-Xylosidase, and Xylose Isomerase Activity

Overnight cultures of the engineered *S. cerevisiae* strains were inoculated into fresh YEPD medium with an initial OD_600_ of 0.2. The cultures were incubated at 30 °C, and samples were withdrawn regularly for enzyme activity assays. To determine the extracellular activity of β-glucosidase and β-xylosidase, the culture broth was centrifuged, and the resulting supernatant was used as the crude enzyme preparation. The protein concentration of the supernatant was measured using an Enhanced BCA Protein Assay Kit (P0010S, Beyotime Biotechnology, Shanghai, China).

The activities of β-glucosidase and β-xylosidase were measured using *p*-nitrophenyl-β-D-glucopyranoside (*p*NPG, Sigma-Aldrich, St. Louis, MO, USA) and *p*-nitrophenyl-β-D-xylopyranoside (*p*NPX, Sigma-Aldrich) as substrates, respectively, following previously reported methods [17,19]. Briefly, 50 μL of 1.0 mg/mL *p*NPG or *p*NPX (dissolved in 50 mM sodium phosphate buffer, pH 4.8) was mixed with 100 μL of appropriately diluted crude enzyme. The reaction mixture was incubated at 50 °C for 30 min, and the reaction was terminated by adding 150 μL of 1 M Na_2_CO_3_. The absorbance at 405 nm was measured, and one unit of enzyme activity was defined as the amount of enzyme that produced 1 μmol *p*-nitrophenol per minute under the assay conditions. 

For the measurement of xylose isomerase activity, a cell-free extract was prepared as the crude enzyme using a Precellys 24-cell homogenizer (Bertin Technologies, Yvelines, France), following a previously described protocol [11,46]. The xylose isomerase activity was determined at 30 °C by monitoring the absorbance change of the coenzymes at 340 nm using a Helios Gamma spectrophotometer (Thermo Fisher Scientific, Waltham, MA, USA). The 1 mL reaction mixture contained 100 mM Tris-HCl buffer (pH 7.5), 10 mM MgCl_2_, 500 mM xylose, 2 U of sorbitol dehydrogenase (Roche Diagnostics GmbH, Mannheim, Germany), 0.15 mM NADH (Sigma-Aldrich, St. Louis, MO, USA), and crude enzyme [47]. One unit of xylose isomerase activity was defined as the amount of enzyme required to oxidize 1 μmol of coenzyme per minute under the assay conditions, and the specific activity was expressed in units per milligram of protein [11,46]. 

### 2.10. Analysis of Hydrolysis and Fermentation Products

The concentrations of glucose, xylose, xylitol, glycerol, acetic acid, and ethanol were determined using high-performance liquid chromatography (HPLC) with an Alliance Separations module e2695 (Waters, Milford, MA, USA). Standard samples of all the above compounds were HPLC-grade and purchased from Sigma-Aldrich. The compounds were separated on an Aminex HPX-87H ion exclusion column (300 × 7.8 mm, Bio-Rad, Richmond, CA, USA) maintained at a temperature of 45 °C. The mobile phase consisted of 5 mM H_2_SO_4_ with a flow rate of 0.6 mL/min. The separated compounds were detected using a Waters 2414 refractive index detector.

The concentrations of cellobiose, xylobiose, and xylotriose were determined using a Dionex ICS-3000 ion chromatography system (Dionex Corporation, Sunnyvale, CA, USA) [12,48,49]. The compounds were separated on a Dionex™ CarboPac™ PA100 IC column (250 × 4 mm) using gradient elution. The column was maintained at an operating temperature of 30 °C to ensure reproducible resolution and retention. The mobile phase consisted of 100 mM NaOH and 500 mM CH_3_COONa with a flow rate of 0.3 mL/min. The separated compounds were detected using an ICS-3000 ED electrochemical detector. The standard sample of cellobiose was purchased from Sigma-Aldrich, and the standard samples of xylobiose and xylotriose were purchased from Shanghai Yuanye Biotechnology Co., Ltd. (Shanghai, China).

## 3. Results and Discussion

### 3.1. Optimization of Protoplast Fusion Operating Conditions

#### 3.1.1. Optimization of Protoplast Preparation and Regeneration Conditions for LF1 and BLN26

Protoplast fusion is a valuable tool for strain improvement in *S. cerevisiae*, enabling the generation of novel strains with desired traits [34,38,50]. The success of protoplast fusion heavily relies on the efficient preparation and regeneration of protoplasts. One of the critical factors influencing the preparation and regeneration efficiency is the concentration of the lytic enzyme used for protoplast preparation, typically Zymolyase [51,52]. In this section, we optimized the conditions for protoplast preparation and regeneration of two *S. cerevisiae* strains, LF1 and BLN26, by evaluating the effect of Zymolyase concentration. 

Zymolyase is a lytic enzyme complex that degrades the yeast cell wall, facilitating the release of protoplasts [53]. However, excessive exposure to Zymolyase can negatively impact the regeneration efficiency of protoplasts. Therefore, it is crucial to optimize the Zymolyase concentration to achieve a balance between high preparation efficiency and satisfactory regeneration efficiency [52,54]. 

Protoplast regeneration requires a high osmotic pressure environment to prevent cell lysis, which can be achieved by supplementing the regeneration medium with osmotic stabilizers such as sorbitol [55]. Sorbitol is a commonly used osmotic stabilizer in protoplast regeneration studies, as it is nontoxic and does not interfere with cell growth. The concentration of 0.8 M sorbitol was chosen based on previous studies demonstrating its effectiveness in supporting protoplast regeneration in *S. cerevisiae* [55].

The effect of Zymolyase concentration on protoplast preparation and regeneration efficiency is shown in Table 3. Reducing the Zymolyase concentration from 5 U/g to 1 U/g (wet cell weight) significantly improved the protoplast regeneration efficiency without compromising the preparation efficiency. However, further reduction of the enzyme concentration to 0.5 U/g led to decreased regeneration efficiency. Notably, at a Zymolyase concentration of 5 U/g, no regenerated protoplasts were detected, indicating that high enzyme concentrations can severely impact regeneration efficiency. Under the optimal Zymolyase concentration of 1 U/g, the protoplast preparation efficiency reached 99.98% and 99.76% for LF1 and BLN26, respectively, while the regeneration efficiency was 42.60% and 54.25%, respectively. These results demonstrate that a balance between high preparation efficiency and satisfactory regeneration efficiency can be achieved by optimizing the Zymolyase concentration.

The protoplast preparation rate and protoplast regeneration rate were calculated using the following formulas:

$$\mathrm{Protoplast}\;\mathrm{Preparation}\;\mathrm{Rate}\;(\%)=\left[\frac{\left(A-C\right)}{A}\right]\times 100\%$$

$$\mathrm{Protoplast}\;\mathrm{Regeneration}\;\mathrm{Rate}\;(\%)=\left[\frac{\left(B-C\right)}{\left(A-C\right)}\right]\times 100\%$$

where A represents the total number of colonies counted on the YEPD solid medium before treatment with Zymolyase; B represents the number of colonies counted on RM medium YPDS after treatment with Zymolyase; and C represents the number of colonies counted on the YEPD solid medium after treatment with Zymolyase.

#### 3.1.2. Optimization of Inactivation Conditions for Protoplasts of LF1 and BLN26

Dual heat and UV inactivation are common technical means for protoplast fusion breeding. Successful protoplast fusion requires the inactivation of parental protoplasts to ensure that only fused protoplasts can regenerate [56]. 

Heat inactivation primarily affects protein synthesis in the cytoplasm, with the ribosomes being the primary target. At present, it is believed that the enzyme protein, 16S ribosomal subunit, and ribosomal RNA in the cytoplasm are damaged after heat treatment of the protoplasm [56]. In contrast, UV inactivation can induce the formation of covalent bonds between adjacent pyrimidine bases on the DNA strand, leading to the creation of dimers, with thymine dimers being the most commonly formed. This results in DNA damage, as the dimers distort the DNA helix, impeding replication and transcription processes [57]. UV radiation can thus compromise the integrity of the genome within the protoplasm, leading to loss of genomic function and cellular inactivation [58]. The regeneration ability of protoplasts caused by the two methods is lost due to damage to different parts. During protoplast fusion, both the nuclei and cytoplasm of the two protoplasts merge, complementing the inactivated components and enabling the regeneration of fused protoplasts [59].

The optimal inactivation conditions for LF1 and BLN26 protoplasts are shown in Table 4. Incubating the protoplasts of LF1 at 60 °C for 15 min resulted in a 100% lethality rate. Exposing the protoplasts of BLN26 to UV light for 7 min resulted in a 100% lethality rate.

The protoplast inactivation rate is calculated using the following formula:

Protoplast inactivation rate (%) = [1 − (A − B)/(C − D)] × 100%,

where A represents the number of colonies observed on RM medium after inactivation, B represents the number of colonies observed on the YEPD solid medium after inactivation, C represents the number of colonies observed on RM medium before inactivation, and D represents the number of colonies observed on the YEPD solid medium before inactivation.

#### 3.1.3. Optimization of Fusion Conditions for Protoplasts of LF1 and BLN26

The success of protoplast fusion depends on optimizing the fusion conditions, which include the concentration of the fusogen (typically polyethylene glycol, PEG), the concentration of divalent cations (such as Ca^2+^), and the fusion time [60,61]. The concentration of PEG plays a crucial role in the fusion process, as it affects the osmotic pressure and the dehydration of protoplast membranes [38]. Divalent cations, such as Ca^2+^ [62], are also essential for protoplast fusion, as they promote membrane adhesion and fusion. The fusion time is another critical factor, as it influences the extent of protoplast contact and fusion events [63]. Orthogonal experimental design is a powerful statistical method for optimizing multiple factors simultaneously while minimizing the number of experiments required. This approach has successfully optimized various bioprocesses, including protoplast fusion in filamentous fungi [62]. In this study, we employed an orthogonal experimental design to investigate the effects of PEG 6000 concentration, CaCl_2_ concentration, and fusion time on the protoplast fusion efficiency of two *S. cerevisiae* parental strains. The factors and levels investigated are shown in Table S1. The R-value was used to determine the main factors influencing the protoplast fusion efficiency and to identify the optimal combination of factors and levels.

Table S1 shows that the concentration of PEG 6000 was the most critical factor influencing the protoplast fusion efficiency, followed by CaCl_2_ concentration and fusion time. Based on the average values of each factor, the optimal fusion conditions were determined to be 30% (*w*/*v*) PEG 6000, 20 mM CaCl_2_, and a fusion time of 20 min.

### 3.2. Screening and Oxygen-Limited Fermentation of Fusants Derived from LF1 and BLN26

#### 3.2.1. Primary Screening of Fusants

In this study, we performed protoplast fusion between *S. cerevisiae* strains LF1 and BLN26 and developed a screening method to select fusants with improved xylose utilization and inhibitor tolerance. The primary screening was conducted in 96-well plates containing YEPD liquid medium, followed by screening on SM agar plates. 

The growth of colonies on solid SM showed significant differences, indicating that SM effectively selects strains with both xylose utilization and tolerance. We selected eight prominent colonies for PCR verification and identified strains BLH01 and BLH02, which possess the characteristic gene sequences for xylose isomerase, β-xylosidase, and β-glucosidase. Subsequently, these strains were subjected to oxygen-limited fermentation to assess their fermentation performances preliminarily. The flowchart for the primary and secondary screening of fusion strains is shown in Figure S2.

#### 3.2.2. Secondary Screening of Fusants

The selected fusants BLH01 and BLH02, along with the parental strains, were subjected to oxygen-limited shake flask fermentation in YPX40, YPD40I2×, and YPX40I_1.5×_ medium to evaluate their metabolic performances. 

**Figure 1:**
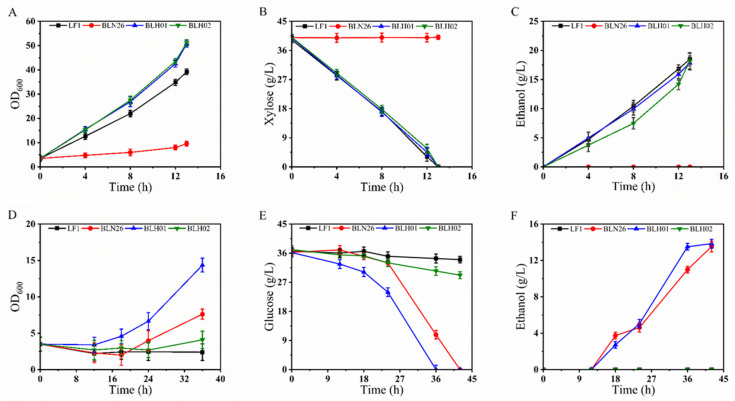
Oxygen-limited fermentation characteristics of strains BLH01, BLH02, LF1, and BLN26 in YPX40 (**A**–**C**) and YPD40I_2×_ (**D**–**F**). The strains were cultured at 30 °C with agitation at 200 rpm in 120 mL serum bottles with a working volume of 40 mL. The fermentation was initiated with a cell density of OD_600_ 3.5, and the oxygen-limited conditions were achieved by sealing the serum bottles with rubber stoppers and inserting a syringe needle to allow limited gas exchange. Data were presented as the means ± standard error of three independent experiments.

Fermentation in YPX40 medium: As shown in Figure 1A, after approximately 13 h of fermentation in the YPX40 medium, the OD_600_ of BLH01 and BLH02 reached approximately 52. Both BLH01, BLH02, and the parental strain LF1 completely consumed the xylose in YPX40 (Figure 1B). BLH01 and BLH02 produced 17.85 g/L and 18.18 g/L ethanol, achieving an ethanol yield of over 87% of the theoretical yield (Figure 1C). In contrast, the parental strain BLN26, which lacks an optimized xylose metabolic pathway, showed negligible growth and did not consume the carbon source xylose (Figure 1B).

Xylose isomerase catalyzes the isomerization of xylose to xylulose, a crucial step in the xylose catabolic pathway. Efficient expression of XI in *S. cerevisiae* is essential for the complete fermentation of xylose, the second most abundant sugar in LCB. The parental strain LF1 is known for its robust xylose metabolism, making it an ideal candidate for developing fusion strains with enhanced fermentation capabilities. Genetic analysis confirmed that the fusion strains BLH01 and BLH02 retained the xylose isomerase gene sequence. Oxygen-limited fermentation results also demonstrated that both fusion strains exhibited superior xylose metabolism, comparable to the parental strain LF1.

Fermentation in YPG40I_2×_ medium: As depicted in Figure 1D, in YPG40I_2×_ medium, strains LF1 and BLH02 exhibited slow growth, utilizing only a tiny amount of glucose. BLN26 consumed all the glucose within 42 h, whereas the fusion strain BLH01 demonstrated the most substantial growth advantage and higher tolerance to complex inhibitors, depleting all the glucose within 36 h (Figure 1E). BLN26 and BLH01 produced 13.526 g/L and 13.84 g/L ethanol, achieving an ethanol yield of 72.9% and 74.9% of the theoretical yield (Figure 1F). Despite the strong inhibitor tolerance exhibited by the parental strain BLN26, the fusion strains BLH01 and BLH02 displayed different levels of robustness. This variation in robustness suggests that genetic exchange and recombination occurred during the protoplast fusion process, highlighting the necessity for screening fusion strains.

**Figure 2:**
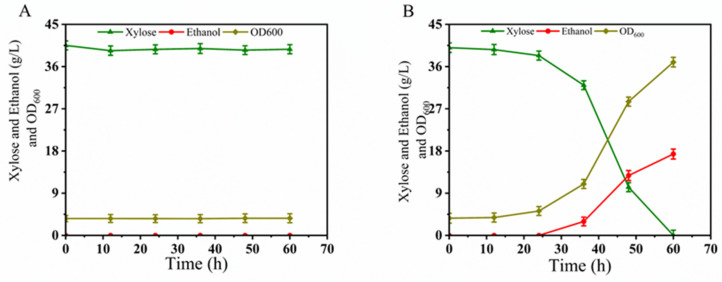
Oxygen-limited fermentation characteristics of strains LF1 (**A**) and BLH01 (**B**) in YPX40I_1.5×_. The strains were cultured at 30 °C with agitation at 200 rpm in 120 mL serum bottles with a working volume of 40 mL. The fermentation was initiated with a cell density of OD_600_ 3.5, and the oxygen-limited conditions were achieved by sealing the serum bottles with rubber stoppers and inserting a syringe needle to allow limited gas exchange. Data were presented as the means ± standard error of three independent experiments.

Fermentation performance evaluation in YPX40I_1.5×_ medium: Subsequently, the fermentation performance of the fusion strain BLH01 was evaluated in YPX40I_1.5×_ medium alongside the parental strain LF1. As shown in Figure 2A, although LF1 had excellent fermentation capacity for xylose in synthetic medium without inhibitors, the presence of the composite inhibitors severely affected the growth of LF1, which could only metabolize a small amount of xylose. In contrast, the fusion strain BLH01 exhibited strong tolerance to inhibitors, completely consuming the xylose within 60 h and producing 17.35 g/L of ethanol, achieving an ethanol yield of over 84.9% of the theoretical yield (Figure 2B). Genomic analysis confirmed that BLH01 retains the characteristic gene sequence for xylose isomerase, essential for xylose metabolism. Fermentation tests demonstrated that BLH01 not only metabolizes xylose efficiently, similar to strain LF1, but also exhibits high tolerance to inhibitors (Figure 2B), akin to strain BLN26 (Figure 1E). These results indicate the successful integration of the desirable traits from both parental strains into BLH01. This integration of traits makes BLH01 a promising candidate for industrial bioethanol production from lignocellulosic biomass, offering enhanced performance under challenging fermentation conditions.

#### 3.2.3. Ploidy Analysis of the Fusant Strain BLH01

Compared to haploid laboratory strains, diploid or polyploid yeast strains typically exhibit higher growth rates, ethanol yields, and tolerance to inhibitors [43,64], making them advantageous for industrial applications [65]. We performed a flow cytometry analysis to confirm the ploidy of the fusion strain BLH01, comparing it with the haploid laboratory strain CEN.PK 102-5B and the diploid parental strain BLN26 [12]. The DNA index (DI) was used to determine the ploidy level of BLH01 [43]. As shown in Figure S3, the DNA content of BLH01 was twice that of the haploid strain CEN.PK 102-5B and consistent with the DNA content of the diploid parental strain BLN26. Therefore, the initial fusion BLH01 is confirmed to be a diploid *S. cerevisiae* strain.

#### 3.2.4. The Genetic Stability Assessment of Fusant BLH01

The genetic stability of the fusant BLH01 was assessed by continuous subculturing in liquid SM medium for multiple generations, followed by subculturing in YEPD medium without selection pressure. This approach is expected to ensure the stable inheritance of desired traits in the fusant and overcome the potential influence of heterokaryosis after protoplast fusion [66]. The heterokaryon phenomenon, which occurs during yeast protoplast fusion, has significant implications for the overall stability of yeast strains. The presence of nuclei from different sources within heterokaryons can lead to genetic instability. For instance, in the study on the protoplast fusion between *S. cerevisiae* and a thermotolerant yeast, the formation of heterokaryons was noted, and it was observed that the resulting fusants exhibited different characteristics compared to their parental strains, indicating potential genetic instability [66]. Research on *Candida albicans* has shown that heterokaryons can be segregant-defective, meaning they may not segregate their genetic material properly during cell division. This can lead to abnormal growth and division, further contributing to genetic instability and affecting the overall stability of the yeast strains [67].

To preliminarily determine whether the superior traits of the fusant strain BLH01 can be stably inherited, we evaluated the fermentation performance of the subcultured strains after multiple rounds of serial subculturing following the abovementioned procedures. As shown in Figure S4, the xylose metabolism capability of the subcultured strains in the YPX40 medium did not significantly differ from that of the parental strain LF1. Both could completely consume 40 g/L of xylose within 15 h of fermentation. In the YPD40I_2×_ medium, the subcultured strains’ fermentation performance continued to surpass that of LF1. The subcultured strains depleted all 40 g/L of glucose within 36 h. In YPX40I_1.5×_ medium, the parental strain LF1 still showed no growth, whereas the subcultured strains of BLH01 could completely consume 40 g/L of xylose within 60 h. These results indicate that the fusion strain BLH01 retains its superior fermentation performance after multiple rounds of serial subculturing.

### 3.3. Selection and Oxygen-Limited Fermentation of Stable Fusants

During the yeast protoplast fusion process, the fusion strain may be genetically unstable, mainly manifested in genetic abnormalities such as chromosome loss and recombination. Maráz highlighted the genetic instability that can arise from recombining and transmitting mitochondrial genes in *S. cerevisiae* after protoplast fusion, including recombination events that may lead to abnormal genetic configurations and potential chromosome loss [68]. Ezeji et al. reported on the production of high-ethanol-yielding yeast strains through protoplast fusion and noted that while some recombinants exhibited enhanced ethanol tolerance and production, others showed significantly lower performance compared to the parental strains. This variability indicates genetic instability, likely due to recombination and potential chromosome loss during fusion [69]. Suzuki et al. reported on chromosome rearrangements and partial chromosome transfer during the protoplast fusion process in *C. albicans*. The fusion derivatives exhibited variations in chromosome size and high-frequency recombination between chromosomes, indicating genetic instability. Chromosome loss was also observed, which can be attributed to spindle abnormalities during mitosis [70]. Lacefield et al. observed that defects in tubulin folding led to abnormal spindle formation and mitotic defects, including abnormal nuclear positioning and shortened spindles. These defects can cause chromosome missegregation and partial chromosome loss, contributing to genetic instability in the yeast strains [71]. Yu et al. investigated the impact of the deletion of the *rpl1001* gene, which encodes a ribosomal protein, on cell mitosis in fission yeast, and found that the deletion resulted in abnormal spindle formation, leading to abnormal chromosome segregation and increased rates of spindle elongation during anaphase. These abnormalities contribute to genetic instability, which can be manifested as partial chromosome loss [72]. 

Although after multiple subcultures, the subcultured strains exhibited fermentation performance similar to the original BLH01 strain, subsequent experiments revealed genetic instability similar to that reported in the above works of literature. This instability manifested as the loss of β-glucosidase and β-xylosidase characteristic gene sequences in some progeny strains, as well as a decrease in xylose fermentation performance in the progeny strains to varying degrees.

#### 3.3.1. Selection of Stable Fusant

Based on the literature review, we hypothesize that the instability of the fusion strain BLH01 may be due to spindle abnormalities during mitosis, leading to partial chromosome loss [71,72]. Consequently, we speculate that BLH01 is more sensitive to mutations affecting homologous recombination, sister chromatid cohesion, and mitotic spindle function. Benomyl, a commercial fungicide, is known to be highly toxic to unstable fusant cells [73]. Therefore, we used benomyl as one of the stress conditions to screen for fusant cells with stable genetic traits. The initial fusion strain BLH01 was subjected to seven rounds of iterative screening. In the first round of screening, BLH01 was plated and screened on YPXB (prepared by supplementing YEPX medium with 20 ng/μL benomyl) solid medium. Approximately 200 large colonies were selected and subjected to secondary screening in a Bioscreen system using YEPX medium supplemented with benomyl (10 ng/μL) and 1.5× mixed inhibitors (15 mM acetic acid, 7.5 mM formic acid, 7.5 mM levulinic acid, 7.5 mM HMF, 7.5 mM furfural, and 7.5 mM vanillin). Based on the growth curves provided by the Bioscreen system, three strains with the best growth advantages were selected for oxygen-limited shake flask fermentation in YPX40I_1.5×_ medium. The strain with the optimal xylose utilization capability was chosen for the second round of iterative screening. The iterative screening process continued for seven rounds. In each round, the survival rate of the fusion strains in the presence of inhibitors was assessed using the Bioscreen system. The strains demonstrating superior growth and inhibitor tolerance were further evaluated for their xylose metabolism in microaerobic shake flask fermentation. After these rounds of iterative screening, we observed a significant reduction in the sensitivity of the fusion strain to benomyl. 

**Figure 3:**
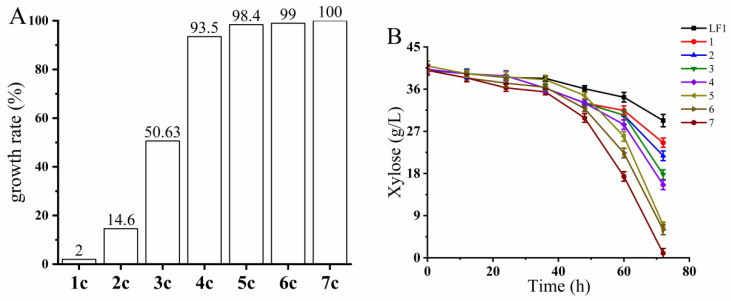
During the iterative screening process, the survival rate of fusion strains in the Bioscreen system (**A**) and the xylose metabolism during oxygen-limited shake flask fermentation (**B**) were monitored. The iterative screening was conducted from the first round (1c) to the seventh round (7c). The initial cell density was OD_600_ = 0.1 for Bioscreen screening (**A**) and OD_600_ = 3.5 for oxygen-limited shake flask fermentation (**B**).

As shown in Figure 3A, the survival rate of the fusant in the Bioscreen system under the combined stress of mixed inhibitors, xylose, and benomyl increased from 2% to 100%. This indicates a marked improvement in the genetic stability of the fusion strain, which also retained good fermentation performance, as shown in Figure 3B. Through the seven rounds mentioned above of iterative screening, we ultimately obtained a robust strain capable of co-fermenting C5 and C6 sugars, named *S. cerevisiae* BLH172. Even after nearly a thousand generations of subculturing, BLH172 maintained stable xylose metabolism in xylose medium containing inhibitors.

#### 3.3.2. Oxygen-Limited Fermentation Performance of BLH172 in YPX40 and YPD40I_2×_

**Figure 4:**
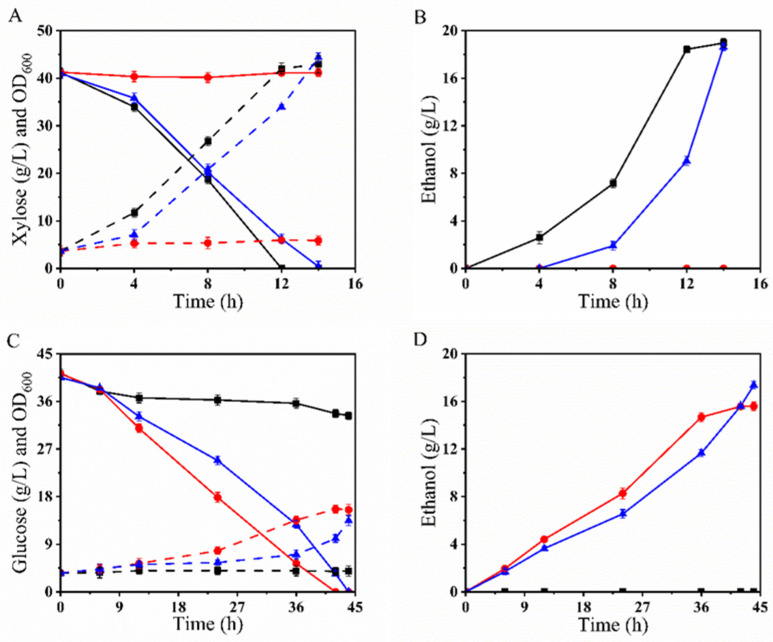
Oxygen-limited fermentation characteristics of strains LF1, BLN26, and BLH172 in YPX40 (**A**,**B**) and YPD40I_2×_ (**C**,**D**). The strains were cultured at 30 °C with agitation at 200 rpm in 120 mL serum bottles with a working volume of 40 mL. The fermentation was initiated with a cell density of OD_600_ 3.5, and the oxygen-limited conditions were achieved by sealing the serum bottles with rubber stoppers and inserting a syringe needle to allow limited gas exchange. Data were presented as the means ± standard error of three independent experiments. Symbols: LF1, ■; BLN26, ●; BLH172, ▲; OD_600_, dotted lines.

Based on the characteristics of the parental strains, we evaluated the fermentation performances and inhibitor tolerance under oxygen-limited conditions using xylose and glucose as carbon sources. The initial OD_600_ was set to 3.5, with a fermentation volume of 40 mL, cultured at 30 °C and 200 rpm. The changes in metabolites and fermentation characteristics during the process are shown in Figure 4.

As shown in Figure 4A,B, during oxygen-limited shake flask fermentation with 40 g/L xylose as the carbon source, the parental strain LF1 demonstrated a significant advantage in xylose utilization, consuming all the xylose within 12 h. The parental strain BLN26, which does not express the Ru-*xylA* gene, could not utilize xylose. The fusion strain BLH172 consumed all the xylose within 14 h, producing 18.63 g/L ethanol, with an ethanol yield of 0.46 g/g, achieving 90% of the theoretical ethanol yield.

As shown in Figure 4C,D, during oxygen-limited shake flask fermentation with YPD40I_2×_ (40 g/L glucose as the carbon source, mixed inhibitors: 20 mM acetic acid, 10 mM formic acid, 10 mM levulinic acid, 10 mM HMF, 10 mM furfural, and 10 mM vanillin), the parental strain LF1 exhibited poor tolerance to the mixed inhibitors and grew slowly. The parental strain BLN26 showed robustness, consuming all the glucose within 42 h. The fusion strain BLH172 consumed all the glucose within 45 h, producing 17.36 g/L ethanol, with an ethanol yield of 0.428 g/g, achieving 83.9% of the theoretical ethanol yield. 

Based on these results, we found that although the fusion strain BLH172 exhibited slightly lower sugar utilization than the parental strains under fermentation conditions, it combined the advantageous traits of both strains. BLH172 demonstrated the xylose utilization advantage of the parental strain LF1 and the robustness of the parental strain BLN26.

#### 3.3.3. Oxygen-Limited Fermentation Performance of BLH172 in YPX40I_1.5×_

**Figure 5:**
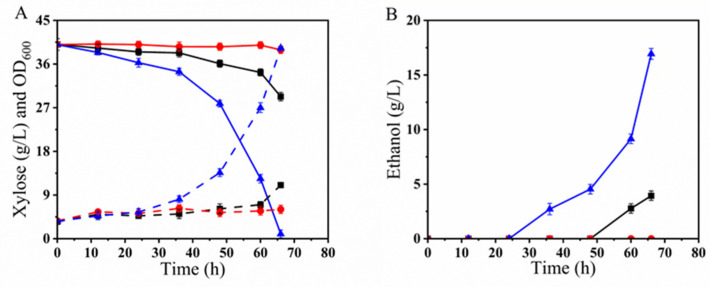
Oxygen-limited fermentation characteristics ((**A**), xylose and OD_600_; (**B**), ethanol) of strains LF1, BLN26, and BLH172 in YPX40I_1.5×_. The strains were cultured at 30 °C with agitation at 200 rpm in 120 mL serum bottles with a working volume of 40 mL. The fermentation was initiated with a cell density of OD_600_ 3.5, and the oxygen-limited conditions were achieved by sealing the serum bottles with rubber stoppers and inserting a syringe needle to allow limited gas exchange. Data were presented as the means ± standard error of three independent experiments. Symbols: LF1, ■; BLN26, ●; BLH172, ▲; OD_600_, dotted lines.

We then conducted oxygen-limited shake flask fermentation using 40 g/L xylose as the carbon source and added 1.5× mixed inhibitors (15 mM acetic acid, 7.5 mM formic acid, 7.5 mM levulinic acid, 7.5 mM HMF, 7.5 mM furfural, and 7.5 mM vanillin). The results are shown in Figure 5A,B. Under these conditions, the parental strains LF1 and BLN26 could not grow (Figure 5A). In contrast, the fusion strain BLH172 could completely utilize the xylose within 66 h, producing 16.93 g/L ethanol (Figure 5B). The ethanol yield was 0.423 g/g, achieving 82.9% of the theoretical yield. The fusion strain BLH172 demonstrated resistance to inhibitors and efficient xylose utilization, highlighting its potential for industrial applications under stress conditions.

### 3.4. Construction of BLH510 with β-Glucosidase and β-Xylosidase Expression Activity

#### 3.4.1. Integration of the β-Glucosidase (BGL) Gene at the *ADH2* Locus

Following PCR verification, it was determined that strain BLH172 had lost the genes for β-glucosidase and β-xylosidase from its chromosomes. However, it retained the xylose isomerase (XI) gene at the δ-sequence and the *PHO13* locus. Alcohol dehydrogenase II (Adh2p), encoded by the *ADH2* gene, catalyzes the oxidation of ethanol to acetaldehyde. Adh2p exhibits an affinity for ethanol that is approximately an order of magnitude higher than that of other alcohol dehydrogenase isozymes [74]. The regulation or disruption of *ADH2* expression has been a key strategy in the metabolic engineering of *S. cerevisiae* [75], particularly in enhancing ethanol production and other metabolic processes [76]. Building on the genetically stable *S. cerevisiae* strain BLH172, we further expressed a heterologous β-glucosidase gene at the *ADH2* locus. As previously described, two homologous arm fragments containing the BGL gene, UP1adh2-BGL-loxP-KanMX-loxP-DOWNadh2 and UP2adh2-BGL-loxP-KanMX-loxP-DOWNadh2, anchored at the *ADH2* locus were prepared using overlapping PCR. The construction process of the integration fragments and the relative positions of the homology arms at the *ADH2* site are shown in Figure S1. These two constructs were sequentially integrated into the genome of strain BLH172, resulting in strain BLH508K, which has the BGL gene integrated at both *ADH2* loci on its chromosomes. Subsequently, the G418 resistance selection marker was removed from the recombinant strain BLH508K using the Cre-loxP recombination system [11,42], and the resulting strain was designated BLH508.

#### 3.4.2. Integration of the β-Xylosidase Gene at the δ-Sequence Locus

Building on the recombinant strain BLH508, we further integrated and expressed a heterologous β-xylosidase gene. The δ-sequence consists of short repetitive sequences on the yeast chromosome, allowing for multi-copy integration via δ-sequence chromosomal homologous recombination [11]. Using this method, we integrated the β-xylosidase cassette IBX (β-xylosidase gene *xyl3A* from *P. oxalicum* with a signal peptide of *Kluyveromyces INU*) into the chromosome of strain BLH508. As previously described, two homologous arm fragments containing the IBX sequence, UP1-IBX-loxP-KanMX-loxP-DOWN and UP2-IBX-loxP-KanMX-loxP-DOWN, anchored at δ-sequence were prepared using overlapping PCR. These two constructs were sequentially integrated into the chromosome of strain BLH508. Transformants were selected and verified by PCR, resulting in the recombinant *S. cerevisiae* strain BLH510K, which heterologously expresses both BGL and IBX. After removing the G418 resistance selection marker, the strain was designated as BLH510.

#### 3.4.3. The Extracellular β-Glucosidase and β-Xylosidase Activities of BLH510

**Figure 6:**
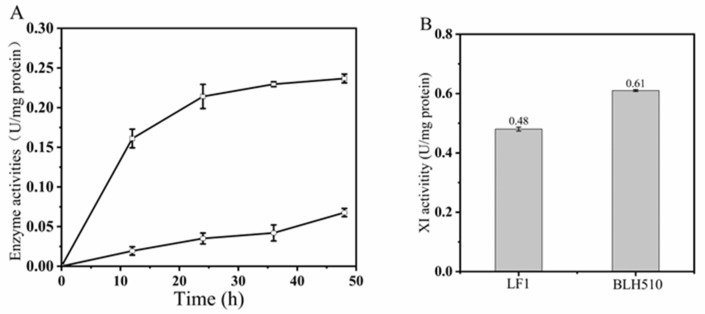
Specific activities of β-glucosidase, β-xylosidase (**A**), and xylose isomerase (**B**) of recombinant *S. cerevisiae* strain BLH510. Data were presented as the means ± standard error of three independent experiments. The symbols: □, β-glucosidase; ○, β-xylosidase.

The extracellular activities of β-glucosidase and β-xylosidase in the engineered strain BLH510 were assessed using *p*NPG and *p*NPX as substrates, respectively. As depicted in Figure 6A, the extracellular activities of both enzymes exhibited an increasing trend throughout the cultivation period. After 48 h of cultivation, the specific extracellular activities of β-glucosidase and β-xylosidase reached 0.236 U/mg protein and 0.067 U/mg protein, respectively. The enzyme activities of strain BLH510 are comparable to those of strain BLN26 [12]. After 48 h of cultivation, the extracellular specific activities of β-glucosidase and β-xylosidase in strain BLN26 were 0.255 U/mg protein (equivalent to 2.18 U/mL) and 0.068 U/mg protein (equivalent to 0.56 U/mL), respectively. These activities were comparatively lower than those reported for previously engineered *S. cerevisiae* strains based on laboratory backgrounds. Tang et al. reported an *S. cerevisiae* haploid strain, 102SB, with a high β-glucosidase secretion activity of 5.2 U/mL, 1.39 times higher than that of BLN26 [17]. Similarly, Niu et al. reported an *S. cerevisiae* haploid strain, BSGIBX, exhibiting a β-xylosidase-specific activity of 6 U/mg protein, which is 88.6 times higher than that of strain BLH510 [19]. Furthermore, Claes et al. reported a recombinant industrial *S. cerevisiae* strain, AC1, with a β-glucosidase activity of 19.1 U/g CDW (approximately 0.525 U/mg protein), and strain AC7 with a β-xylosidase activity of approximately 0.341 U/mg protein [77]. As shown in Figure 6B, the specific activity of xylose isomerase in strain BLH510 (0.61 U/mg protein) increased by 27% compared to the parental strain LF1.

### 3.5. Oxygen-Limited Fermentation of BLH510

#### 3.5.1. Fermentation of BLH510 with Cellobiose and the Mixture of Glucose–Cellobiose or Xylose–Cellobiose

**Figure 7:**
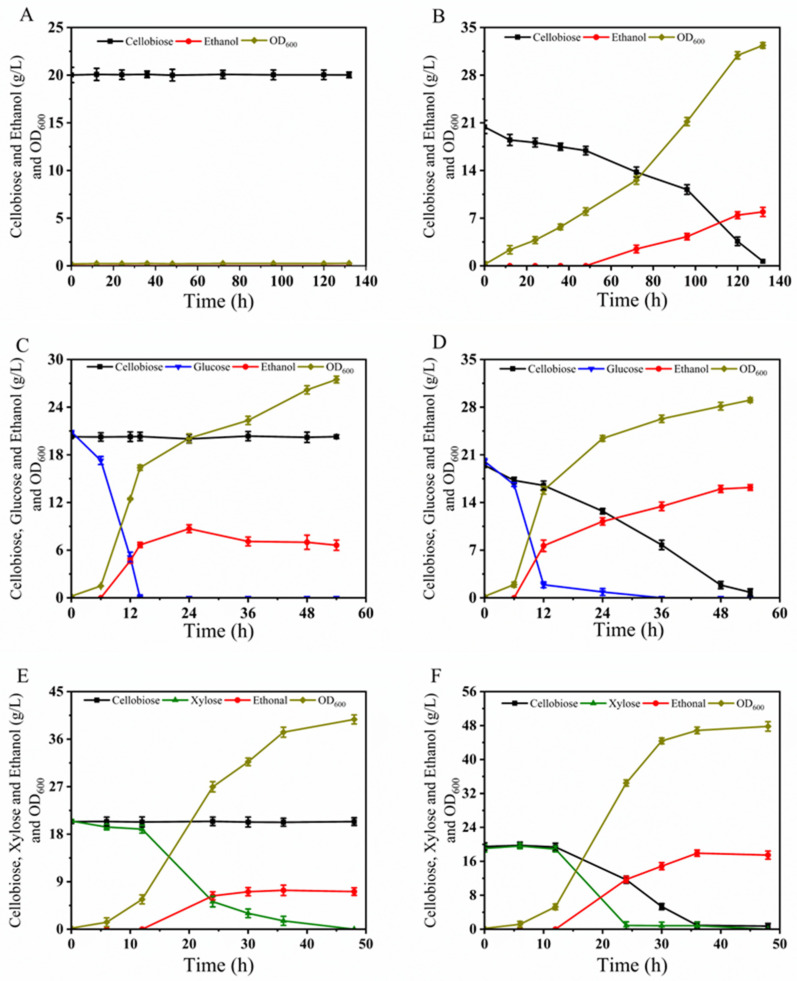
Oxygen-limited fermentation characteristics of strains BLH172 (**A**,**C**,**E**) and BLH510 (**B**,**D**,**F**) in shake flasks. The experiments were performed in triplicate in YP (10 g/L yeast extract and 20 g/L tryptone) with cellobiose (YPC, (**A**,**B**)), glucose–cellobiose mixture (YPGC, (**C**,**D**)), and xylose–cellobiose mixture (YPXC, (**E**,**F**)). The strains were cultured at 30 °C with agitation at 200 rpm in 120 mL serum bottles with a working volume of 40 mL. The fermentation was initiated with a cell density of OD_600_ 0.2, and the oxygen-limited conditions were achieved by sealing the serum bottles with rubber stoppers and inserting a syringe needle to allow limited gas exchange. Data were presented as the means ± standard error of three independent experiments.

Strain BLH510 exhibits extracellular β-glucosidase activity. We subsequently conducted oxygen-limited shake flask fermentation tests using cellobiose as the sole carbon source. As shown in Figure 7A, strain BLH172, which lacks the β-glucosidase coding gene in its genome, could not grow on cellobiose. In contrast, strain BLH510 successfully integrated the β-glucosidase gene at the *ADH2* locus in its genome. As depicted in Figure 7B, under conditions with no other carbon source, strain BLH510 completely consumed the cellobiose in the medium after approximately 132 h of fermentation, producing about 7.92 g/L ethanol. This indicates that through metabolic engineering, strain BLH510 has indeed been endowed with the ability to ferment cellobiose, demonstrating its potential for industrial applications involving cellobiose utilization. 

In the YPGC medium, where glucose and cellobiose were used as co-carbon sources, the cellobiose metabolism rate of strain BLH510 significantly increased. As a control, even in the presence of glucose, BLH172 could metabolize glucose but still could not utilize cellobiose as a carbon source (Figure 7C). As shown in Figure 7D, strain BLH510 could completely consume both glucose and cellobiose within 54 h of fermentation. The fermentation process produced 16.21 g/L ethanol, with an ethanol yield of 0.42 g/g, achieving 82.2% of the theoretical ethanol yield.

In the YPXC medium, where xylose and cellobiose are used as co-carbon sources, strain BLH510 exhibited further enhanced cellobiose metabolism and demonstrated co-fermentation of xylose and cellobiose. As shown in Figure 7F, strain BLH510 could completely consume all the sugars within approximately 36 h. The fermentation process resulted in the production of 17.48 g/L ethanol, with an ethanol yield of 0.45 g/g, achieving 88.3% of the theoretical ethanol yield. In contrast, under the same conditions, the presence of cellobiose slowed down the xylose metabolism of strain BLH172, which took about 48 h to consume all 20 g/L of xylose in the YPXC medium(Figure 7E). These findings indicate that strain BLH510 not only has an improved ability to metabolize cellobiose but also effectively co-ferments glucose/cellobiose and xylose/cellobiose, making it a promising candidate for efficient bioethanol production from mixed sugar substrates.

#### 3.5.2. Fermentation of BLH510 with the Mixture of Glucose–Xylose–Cellobiose

**Figure 8:**
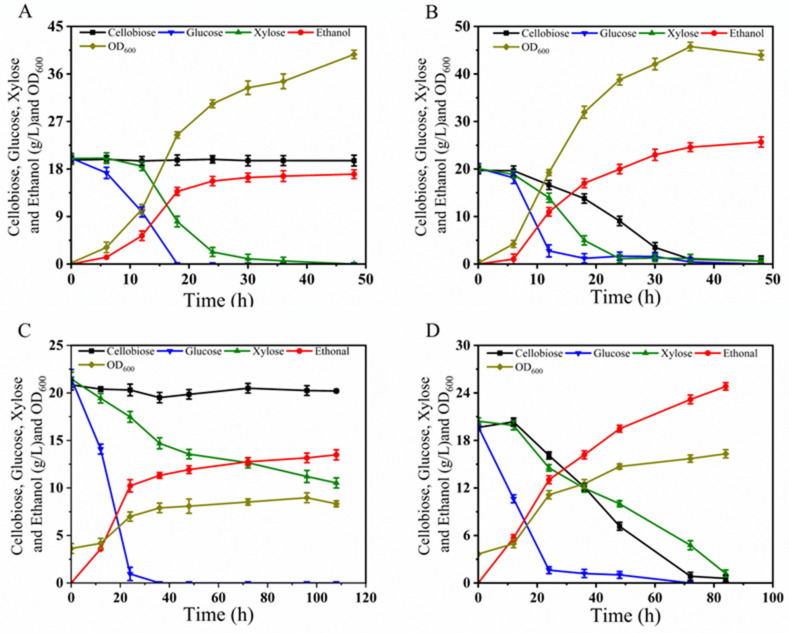
Oxygen-limited fermentation characteristics of strains BLH172 (**A**,**C**) and BLH510 (**B**,**D**) in shake flasks. The experiments were performed in triplicate in YP (10 g/L yeast extract and 20 g/L tryptone) with glucose–xylose–cellobiose mixture (YPGXC, (**A**,**B**)) and glucose–xylose–cellobiose-inhibitors mixture (YPGXCI_1.5×_, (**C**,**D**)). The strains were cultured at 30 °C with agitation at 200 rpm in 120 mL serum bottles with a working volume of 40 mL. The fermentation was initiated with a cell density of OD_600_ 0.2 (**A**,**B**) or OD_600_ 3.5 (**C**,**D**), and the oxygen-limited conditions were achieved by sealing the serum bottles with rubber stoppers and inserting a syringe needle to allow limited gas exchange. Data were presented as the means ± standard error of three independent experiments.

We evaluated the fermentation capability of strain BLH510 in YPGXC medium, which contains a mixed carbon source of 20 g/L glucose, 20 g/L xylose, and 20 g/L cellobiose. As shown in Figure 8A, strain BLH172 demonstrated efficient co-fermentation of glucose and xylose but could not metabolize cellobiose. In contrast, the recombinant strain BLH510 retained the co-fermentation capabilities of its parental strains for glucose and xylose and successfully co-fermented glucose, xylose, and cellobiose. Within approximately 36 h, BLH510 nearly completely utilized all the sugars, producing 23.89 g/L ethanol, with an ethanol yield of 0.437 g/g, achieving 85.6% of the theoretical ethanol yield (Figure 8B).

To further validate the inhibitor tolerance of strain BLH510, we conducted oxygen-limited shake flask fermentation in YPGXC medium supplemented with 1.5× inhibitor mixture (YPGXCI_1.5×_, 15 mM acetic acid, 7.5 mM formic acid, 7.5 mM levulinic acid, 7.5 mM HMF, 7.5 mM furfural, and 7.5 mM vanillin) mixed inhibitors, starting with an initial OD_600_ of 3.5. As shown in Figure 8C, in the presence of inhibitors, strain BLH172 could completely consume glucose; however, approximately half of the xylose remained unutilized after 108 h of fermentation. In contrast, strain BLH510 could completely consume all the sugars within approximately 84 h, producing 24.82 g/L ethanol, with an ethanol yield of 0.428 g/g, achieving 83.9% of the theoretical ethanol yield (Figure 8D). 

#### 3.5.3. Fermentation of BLH510 with the Mixture of Glucose–Xylose–Cellobiose-XOS_pre_

To maximize the utilization of XOS, a pretreatment step using xylanase was employed. The XOS substrate was treated with xylanase (kindly provided by Qingdao Vland Biotech Inc., Qingdao, China) at an enzyme dosage of 3 mg/g of XOS. The xylanase exhibited an activity of approximately 170 U/mg protein. The enzymatic pretreatment was carried out at 50 °C for 36 h, allowing for the efficient hydrolysis of the XOS components [12,19]. The thin-layer chromatography of the products after enzymatic hydrolysis of XOS is shown in Figure S5, which mainly consists of xylobiose and xylose with a lower degree of polymerization and a low amount of xylotriose.

**Figure 9:**
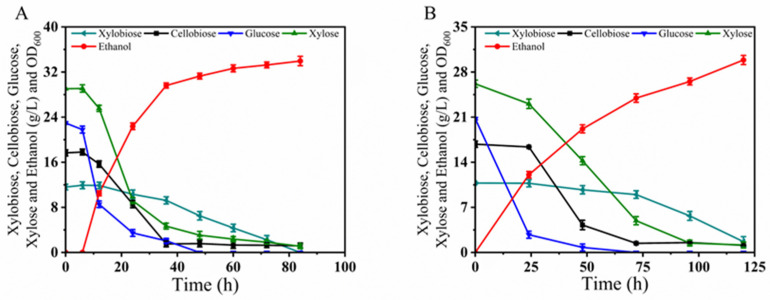
Oxygen-limited fermentation characteristics of BLH510 in the media of YPGXCO_pre_ and YPGXCO_pre_I_1.5×_. The experiments were performed in YP medium supplemented with glucose–xylose–cellobiose–XOS_pre_ mixture (YPGXCO_pre_, (**A**)) and glucose–xylose–cellobiose–XOS_pre_–inhibitors mixture (YPGXCO_pre_I_1.5×_, (**B**)). The strains were cultured at 30 °C with agitation at 200 rpm in 120 mL serum bottles with a working volume of 40 mL. The initial cell density was set to OD_600_ = 0.2 (**A**) or OD_600_ = 3.5 (**B**), and oxygen-limited conditions were maintained using a rubber stopper plug with a syringe needle. Data were presented as the means ± standard error of three independent experiments.

The fermentation medium YPGXCO_pre_, as shown in Figure 9, was composed of a mixture of glucose, xylose, cellobiose, and pre-hydrolyzed XOS (XOS_pre_). Due to the integration of the β-xylosidase gene into the δ-sequence of its chromosome, strain BLH510 exhibits efficient metabolism of xylobiose. As shown in Figure 9A, during fermentation, BLH510 nearly depleted the glucose, xylose, and cellobiose in the medium within 48 h. By 84 h, all the xylobiose was also metabolized, producing 33.96 g/L ethanol, with an ethanol yield of 0.43 g/g, achieving 84.3% of the theoretical ethanol yield. Under conditions with 1.5× inhibitor mixture (YPGXCO_pre_I_1.5×_, 15 mM acetic acid, 7.5 mM formic acid, 7.5 mM levulinic acid, 7.5 mM HMF, 7.5 mM furfural, and 7.5 mM vanillin), as shown in Figure 9B, BLH510 nearly depleted the glucose, xylose, and cellobiose in the medium within 96 h. By 120 h, all the xylobiose was also metabolized. These results demonstrate that integrating the β-xylosidase gene significantly enhances the ability of strain BLH510 to metabolize xylobiose. Strain BLH510 efficiently utilizes mixed sugars, including glucose, xylose, cellobiose, and xylobiose, even in the presence of inhibitors, making it a robust candidate for bioethanol production from complex lignocellulosic hydrolysates, resulting in the production of 29.87 g/L ethanol, with an ethanol yield of 0.423 g/g, achieving 82.9% of the theoretical ethanol yield.

### 3.6. Determination of Intracellular Trehalose Content in S. cerevisiae Strains

**Figure 10:**
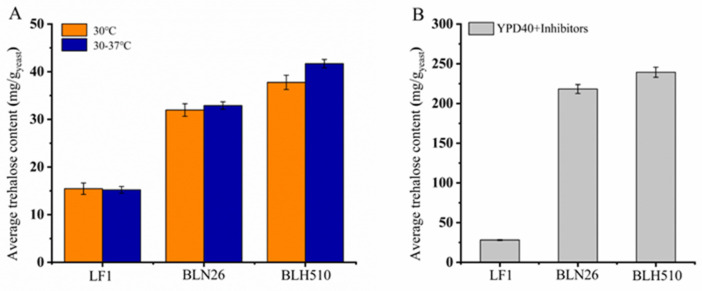
Intracellular trehalose content of *S. cerevisiae* strains LF1, BLN26, and BLH510 in YPD40 (**A**) and YPD40I_2×_ (**B**). The trehalose content was quantified using the anthrone–sulfuric acid method. Data were presented as the means ± standard error of three independent experiments.

Trehalose is a storage carbohydrate and a vital stress metabolite that enhances survival by protecting organisms or cells from damage under adverse conditions such as drought, dehydration, high temperature, freezing, high osmotic pressure, and exposure to toxic agents [44]. The trehalose content of *S. cerevisiae* strains was measured, as shown in Figure 10. 

As depicted in Figure 10A, the strains were cultured under oxygen-limited conditions with 40 g/L glucose as the sole carbon source. At normal cultivation temperature (30 °C), the parental strains LF1 and BLN26 exhibited lower trehalose content compared to the fusion strain BLH510. During a gradual temperature increase from 30 °C to 37 °C at 21-min intervals, the trehalose content in LF1 and BLN26 remained unchanged. In contrast, BLH510 demonstrated a significant increase in trehalose content, reaching an intracellular concentration of 41.7 mg/g yeast, indicating its enhanced tolerance to external temperature stress.

Figure 10B illustrates the strains cultured under oxygen-limited conditions in YPD40I_2×_ medium, a medium containing 40 g/L glucose as the sole carbon source and 2× inhibitor mixture (20 mM acetic acid, 10 mM formic acid, 10 mM levulinic acid, 10 mM HMF, 10 mM furfural, and 10 mM vanillin). 

Under these fermentation conditions, the parental strain LF1 exhibited a very low trehalose content of approximately 25.0 mg/g yeast, likely due to its low inhibitor tolerance. In comparison, BLH510 displayed a higher trehalose content than the parental strain BLN26, with an intracellular concentration reaching 239.3 mg/g yeast. This trehalose content exceeds 20% of the dry weight, suggesting that BLH510 possesses the potential for active dry yeast production [78]. 

The ability of BLH510 to accumulate high levels of trehalose under both temperature stress and inhibitor-rich conditions highlights its superior stress tolerance compared to its parental strains. This characteristic is particularly valuable for the efficient fermentation of lignocellulosic hydrolysates, which often contain various inhibitory compounds that can negatively impact yeast performance. The capacity of BLH510 to maintain elevated trehalose levels in the presence of these stressors underscores its potential as a robust strain for second-generation bioethanol production and active dry yeast manufacturing.

### 3.7. Determination of the Ratio of Reduced and Oxidized Glutathione (GSH/GSSG)

**Figure 11:**
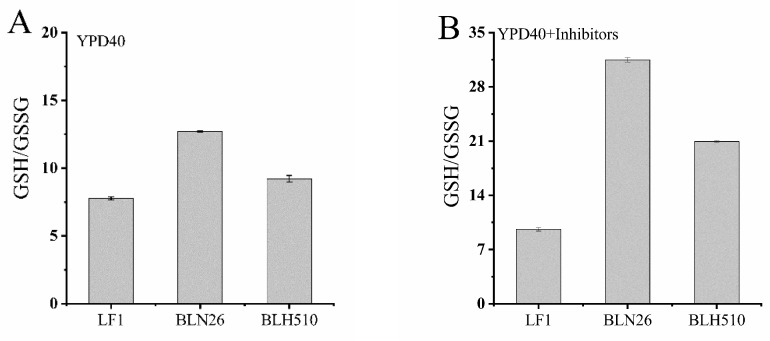
Glutathione content (the ratio of cellular GSH to GSSG) of *S. cerevisiae* strains LF1, BLN26, and BLH510 in YPD40 (**A**) and YPD40I_2×_ (**B**). The GSH and GSSG Assay Kit (S0053, Beyotime Biotechnology, Shanghai, China) was used for GSH/GSSG determination. Data were presented as the means ± standard error of three independent experiments.

GSH is a crucial cellular protectant that serves as an endogenous antioxidant in various metabolic functions of eukaryotic cells. In *S. cerevisiae*, GSH is synthesized in the cytosol through two ATP-dependent steps, and yeast strains deficient in GSH are susceptible to oxidative stress. Industrial production of GSH typically involves the fermentation of *S. cerevisiae* with glucose. Thus, we selected the YPD40 medium suitable for yeast growth for oxygen-limited shake flask culture. Under conditions where 40 g/L glucose was the sole carbon source, and no inhibitors were added, the GSH/GSSG ratios of the three yeast strains did not differ significantly (Figure 11A).

However, furfural and 5-hydroxymethyl furfural, known inhibitors in lignocellulosic hydrolysates, are thiol-reactive electrophiles that deplete GSH levels in *S. cerevisiae*. When the GSH content of the strains was measured in a YPD40 liquid medium containing mixed inhibitors (YPD40I_2×_), strain LF1 exhibited a low GSH/GSSG ratio (below 10) due to its poor tolerance to the inhibitors, resulting in minimal growth. In contrast, strain BLN26, which has a higher tolerance to the inhibitors, showed a significant increase in glutathione production, with a GSH/GSSG ratio of 31.5. The fusion strain BLH510, which combines the xylose utilization and inhibitor tolerance advantages of both parents, achieved a GSH/GSSG ratio of 21 (Figure 11B), indicating that it possesses a certain degree of resistance to oxidative stress.

## 4. Conclusions

Developing robust and efficient *S. cerevisiae* strains is crucial for economically viable second-generation bioethanol production from lignocellulosic biomass. In this study, protoplast fusion was successfully employed to generate a novel *S. cerevisiae* strain, BLH510, that combines the advantageous traits of its parental strains, LF1 and BLN26. Through rigorous screening and metabolic engineering, BLH510 demonstrated efficient co-fermentation of mixed sugars (glucose, xylose, cellobiose, and XOS) and enhanced tolerance to inhibitors commonly found in lignocellulosic hydrolysates.

The optimization of protoplast preparation and fusion conditions played a critical role in the success of this study. A high protoplast preparation efficiency and satisfactory regeneration efficiency were achieved by carefully balancing the Zymolyase concentration. Furthermore, the orthogonal experimental design employed to investigate the effects of PEG 6000 concentration, CaCl_2_ concentration, and fusion time on the protoplast fusion efficiency ensured the identification of optimal fusion conditions.

The iterative screening process involved using benomyl as a selective agent and was instrumental in obtaining a genetically stable fusion strain, BLH172. The subsequent integration of β-glucosidase and β-xylosidase genes into BLH172 resulted in the final strain, BLH510, which exhibited efficient hydrolysis and fermentation of cellobiose and xylooligosaccharides. The enhanced stress tolerance of BLH510, as evidenced by its increased intracellular trehalose content and glutathione ratio under challenging conditions, highlights its potential for industrial applications.

In conclusion, this study demonstrates the successful development of a robust *S. cerevisiae* strain, BLH510, through protoplast fusion and metabolic engineering. The strain’s ability to efficiently co-ferment mixed sugars and tolerate inhibitors makes it a promising candidate for second-generation bioethanol production from lignocellulosic biomass.

## Figures and Tables

**Table 2:** Primers used in this work.

Name	Primes Sequence (5′→3′)	Description
BGL-F	AGATCTACGTATGGACTTTCTTCTTCAG	BGL
BGL-R	AAGGGTTGTCGACCTGCAGCTATTCCTTCAATTCAGCAACAAAATTTGAA	
loxP-F	GCTGCAGGTCGACAACCCTTAAT	KanMX
loxP-R	GGCCACTAGTGGATCTGATATCACC	
XYL-F	GGAAGTACCTTCAAAGAATGGGGTC	IBX
XYL-R	GGGTTGTCGACCTGCAGCCTTCGAGCGTCCCAAAACCTTC	
U1-ADH2-F	GAAGGTGCCGGTGTCGTT	UP1 *adh2*
U1-ADH2-R	GCAAATACGTAGATCTGCGTCAGCGGTAGCGTATt	
D-ADH2-F	CCACTAGTGGCCGGCTACCAACGGCGGT	DOWN *adh2*
D-ADH2-R	CAGCTCTGTTCCCCACGTAAGA	
U2-ADH2-F	CAATCTTGTGTGCTGGTATCACCG	UP2 *adh2*
U2-ADH2-R	GACCATACGTAGATCTTTCACCACCGAGCGAGGTAAA	
UP1-δ-F	TATGTATTTTTAATCGTCCTTGTATGGAAGTATCAAAGG	δ 1
UP1-δ-R	GACCCCATTCTTTGAAGGTACTTCCCAACAACCTCTTGCTATCAAACTTACTTTTG	
D-δ-F	GGTGATATCAGATCCACTAGTGGCCGGCTTCATTGTAACATGTAAGTGAACATC	δ 2
D-δ-R	CAGGCTTATAATGTCAGTATGCATTGTTACG	
UP2-δ-F	AACGTTAAAAGAGTAGCTCAGACAGTG	δ 3
UP2-δ-R	GACCCCATTCTTTGAAGGTACTTCCGAATATGGATCTTAATATAATCGTATAAGAGGGGC	

**Table 3:** Effect of Zymolyase concentration on protoplast preparation and regeneration.

Zymolyase Concentration (U/g Wet Cell Weight)	Protoplast Preparation Rate		Protoplast Regeneration Rate	
	LF1	BLN26	LF1	BLN26
5	99.99%	99.99%	Not detected	Not detected
3	99.99%	99.99%	0.09%	1.219%
2	99.95%	99.85%	12.50%	22.07%
1	99.98%	99.76%	42.60%	54.25%
0.5	91.97%	90.5%	40.21%	45.82%

**Table 4:** Lethality Rates for Heat and UV Treatments on LF1 and BLN26.

Heat treatment (min)	2	5	10	15	20
lethality rate of LF1 (%)	15.8 ± 0.01	90.1 ± 0.02	98.5 ± 0.00	100 ± 0.00	100 ± 0.00
UV treatment (min)	1	2	5	7	10
lethality rate of BLN26 (%)	56.3 ± 0.02	87.5 ± 0.01	95.4 ± 0.02	100 ± 0.00	100 ± 0.00

## Data Availability

The original contributions presented in the study are included in the article/Supplementary Material, further inquiries can be directed to the corresponding author.

## Supplementary Materials

The following supporting information can be downloaded at: https://www.mdpi.com/article/10.3390/microorganisms12081526/s1, Table S1: Orthogonal experimental design and results for optimizing protoplast fusion conditions; Figure S1: The construction process of the integration fragments and the relative positions of the homology arms at the *ADH2* site; Figure S2: Primary and secondary screening of fusion strains; Figure S3: Determination of DNA content by flow cytometry of *S. cerevisiae* strains; Figure S4: Oxygen-limited fermentation characteristics of LF1 (a,c,e) and subcultured strains of BLH01 (b,d,f) in YPX40 (a,b), YPD40I_2×_ (c,d), and YPX40I_1.5×_ (e,f); Figure S5: The thin-layer chromatography of the products after enzymatic hydrolysis of XOS.
