# Supplementary material for: Development of a Robust Saccharomyces cerevisiae Strain for Efficient Co-Fermentation of Mixed Sugars and Enhanced Inhibitor Tolerance through Protoplast Fusion
Source: Microorganisms. 2024 Jul 25;12(8):1526. doi: 10.3390/microorganisms12081526 (PMC11356107; doi:10.3390/microorganisms12081526)
Supplement: Supplementary file 1 [file microorganisms-12-01526-s001.zip › microorganisms-3119366-supplementary.pdf]

Table S1. Orthogonal experimental design and results for optimizing protoplast fusion conditions.

Level	Factors			Fusion rate (%)
	PEG concentration (%)	$CaCl_2$ concentration (mM)	T (min)	
1	30	5	10	0.0345
2	30	10	15	0.058
**3**	**30**	**20**	**20**	**0.122**
4	35	5	10	0.087
5	35	10	15	0.054
6	35	20	20	0.051
7	40	5	10	0.064
8	40	10	15	0.081
9	40	20	20	0.104
T1	0.166	0.186	0.216	-
T2	0.250	0.294	0.292	-
T3	0.341	0.277	0.250	-
K1	0.056	0.062	0.072	-
K2	0.083	0.098	0.097	-
K3	0.114	0.092	0.083	-
R	0.058	0.036	0.026	-

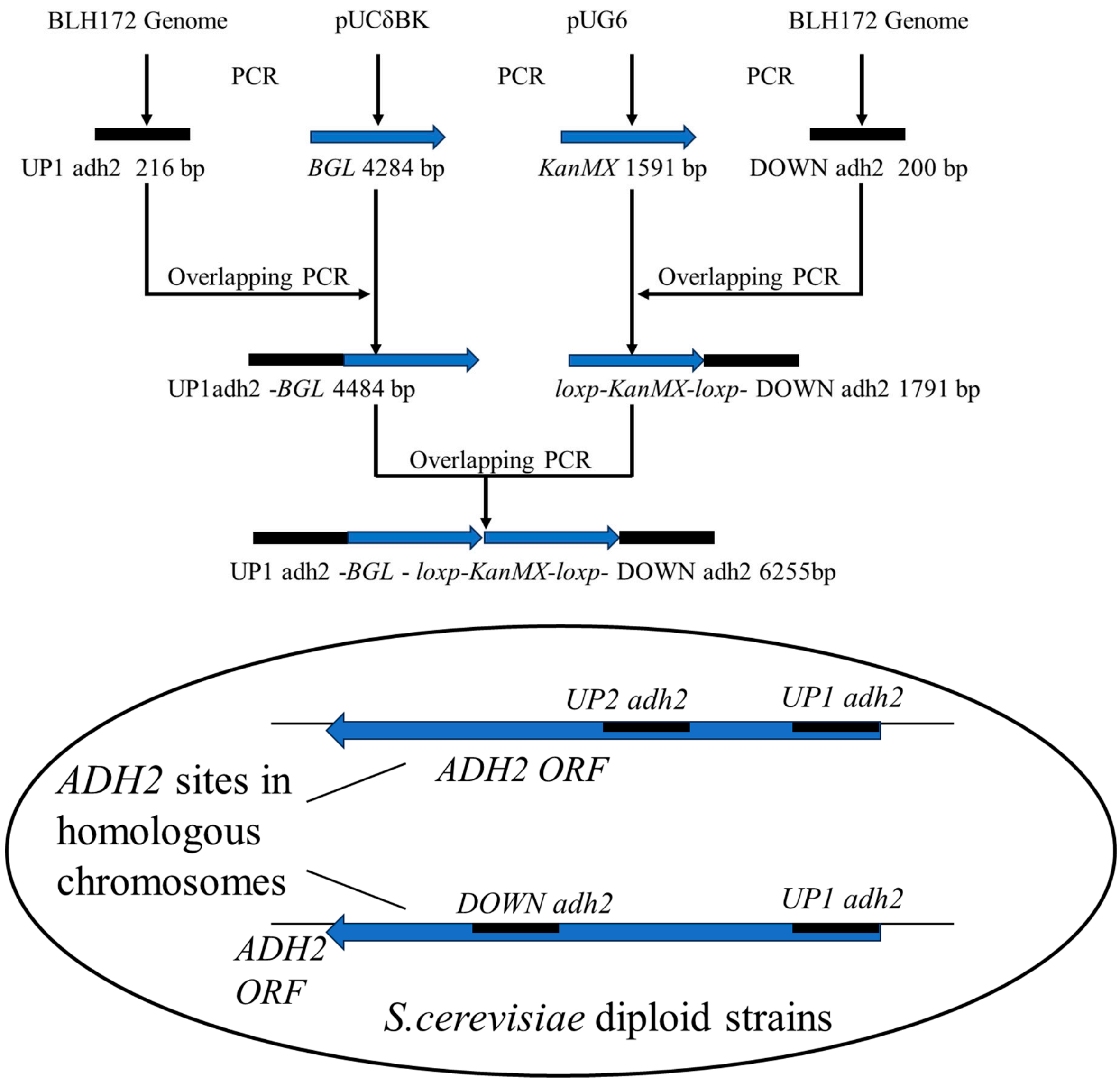

Figure S1. The construction process of the integration fragments and the relative positions of the homology arms at the *ADH2* site.

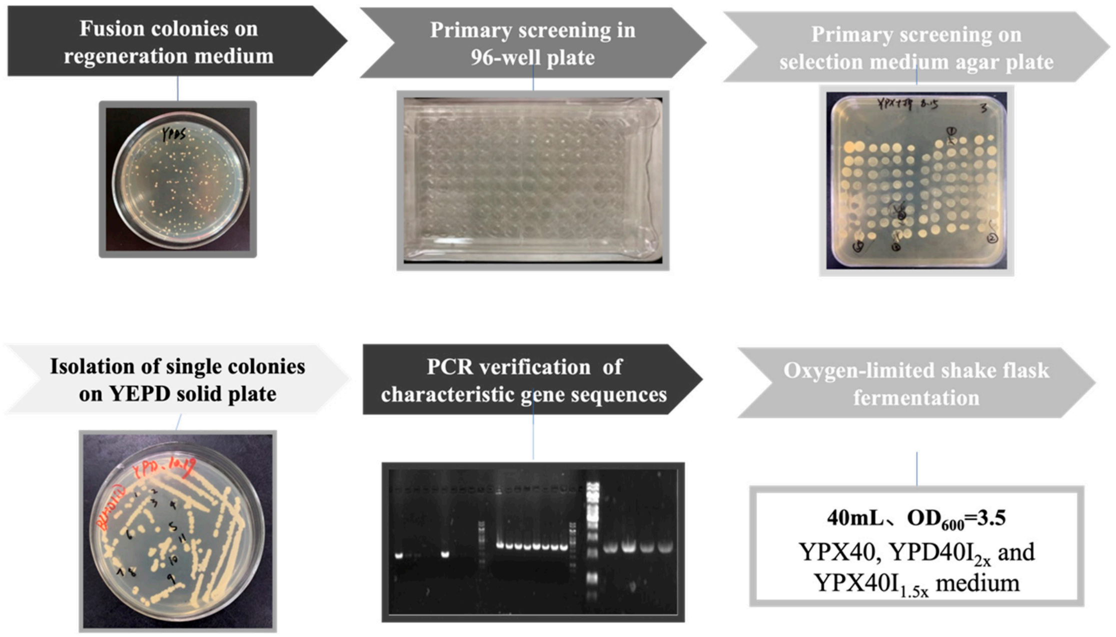

Figure S2. Primary and secondary screening of fusion strains.

The protoplast regeneration medium (RM) was prepared by supplementing YEPX solid medium with 0.8 M sorbitol to maintain a high osmotic pressure environment. The YPXB solid medium was prepared by supplementing YEPX solid medium with 20 ng/μL benomyl. The medium was sterilized by autoclaving at 115°C for 30 min. The selection medium (SM) was based on YEPX and supplemented with various inhibitory compounds, including 10 mM acetic acid, 5 mM formic acid, 5 mM levulinic acid, 5 mM furfural, 5 mM HMF, and 5 mM vanillin, to facilitate the selection of hybrids with improved tolerance to these stressors

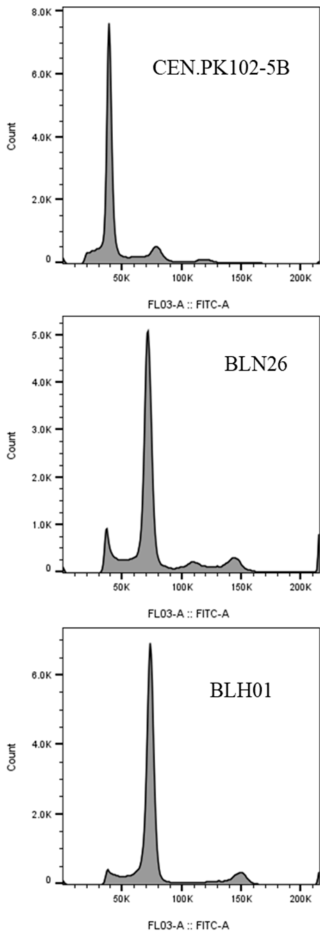

Figure S3. Determination of DNA content by flow cytometry of *S. cerevisiae* strains.

Strains were grown to exponential phase then fixed with ethanol, and the DNA was stained with propidium iodide. The X-coordinate FL03A represents total cell fluorescence, and it is proportional to DNA content. The Gaussian curves in density plot represent the G0/G1 phase of cells. The strain ploidy was then determined by the G0/G1 peak value on FL03A versus that of the haploid strain.

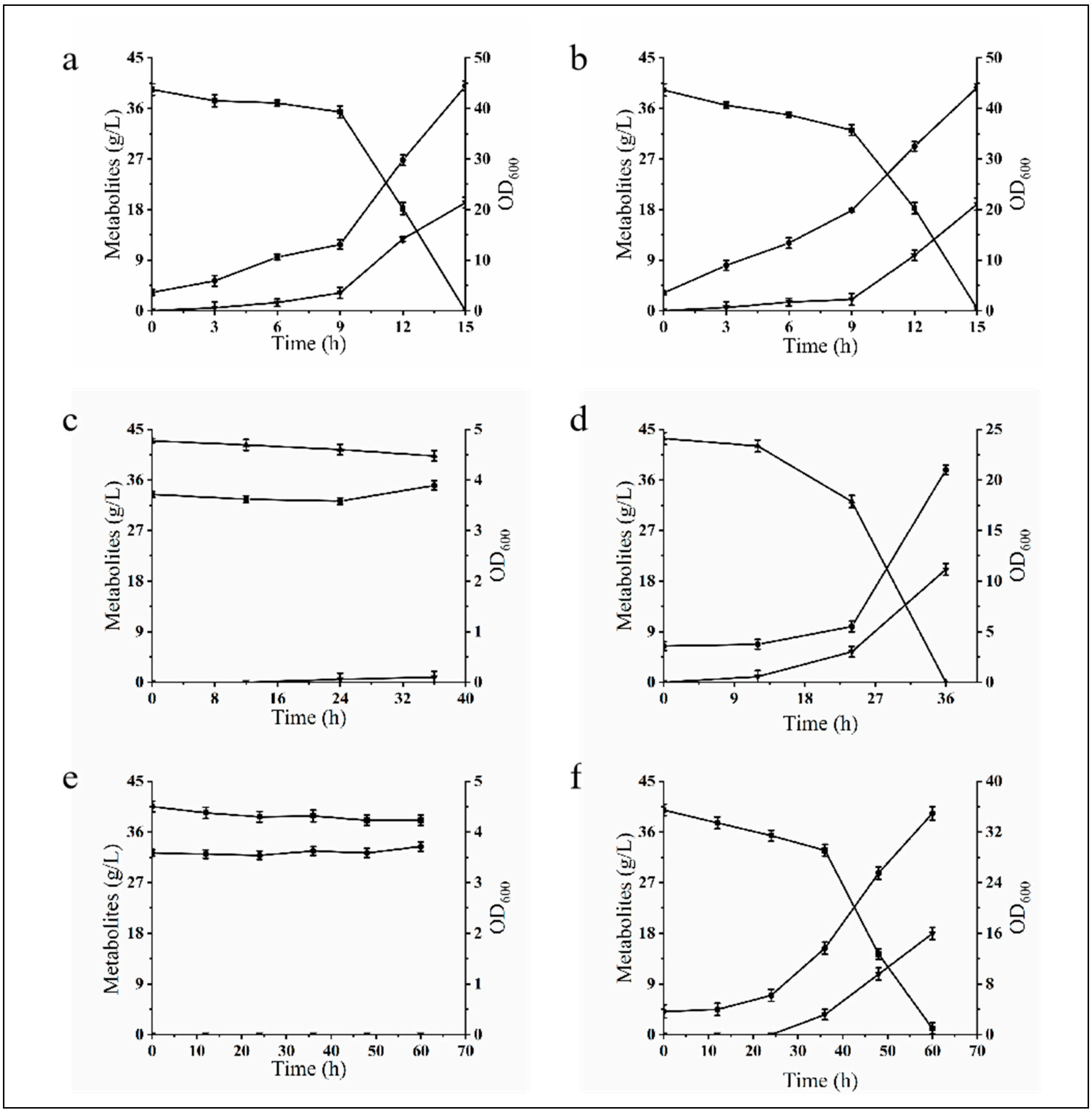

Figure S4. Oxygen-limited fermentation characteristics of LF1 (a, c, e) and subcultured strains of BLH01 (b, d, f) in YPX40 (a, b), YPD40I$_{2x}$ (c, d) and YPX40I$_{1.5x}$ (e, f).
The initial cell density was $OD_{600} = 3.5$, and the oxygen-limited condition was provided by a rubber stopper plug in a syringe needle. Each experimental group was performed in triplicate. The concentrations of mixed inhibitors in YPD40I$_{2x}$ were 20 mM acetic acid, 10 mM formic acid, 10 mM levulinic acid, 10 mM furfural, 10 mM HMF and 10 mM vanillin; The concentrations of mixed inhibitors in YPX40I$_{1.5x}$ were 15 mM acetic acid, 7.5 mM formic acid, 7.5 mM levulinic acid, 7.5 mM furfural, 7.5 mM HMF and 7.5 mM vanillin. Symbols: Xylose, ■; Glucose▲; OD_{600}, ●; Ethanol, ▼;

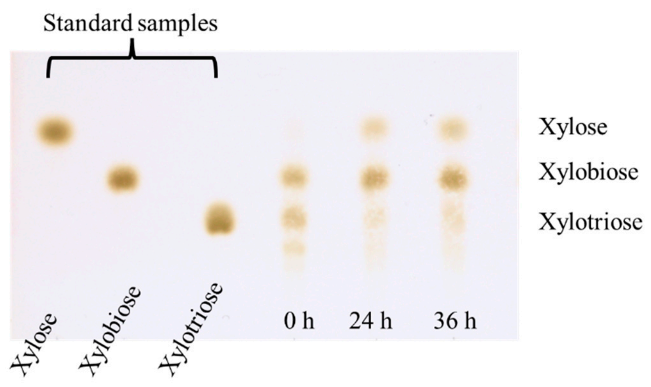

Figure S5. The thin layer chromatography of the products after enzymatic hydrolysis of XOS.
Xylanase (with the activity of approximately 170 U mg^{-1} protein, kindly provided by Qingdao Vland Biotech Inc., Qingdao, China) was added to pretreat XOS for 36 h at 50 °C with an enzyme dosage of 3 mg g^{-1} XOS
